# Comparative transcriptomic analysis of dermal wound healing reveals *de novo* skeletal muscle regeneration in *Acomys cahirinus*

**DOI:** 10.1371/journal.pone.0216228

**Published:** 2019-05-29

**Authors:** Jason O. Brant, J. Lucas Boatwright, Ruth Davenport, Aaron Gabriel W. Sandoval, Malcolm Maden, W. Brad Barbazuk

**Affiliations:** 1 Department of Biology, University of Florida, Gainesville, Florida, United States of America; 2 Genetics Institute, University of Florida, Gainesville, Florida, United States of America; University of Minnesota Medical Center, UNITED STATES

## Abstract

The African spiny mouse, *Acomys* spp., is capable of scar-free dermal wound healing. Here, we have performed a comprehensive analysis of gene expression throughout wound healing following full-thickness excisional dermal wounds in both *Acomys cahirinus* and *Mus musculus*. Additionally, we provide an annotated, *de novo* transcriptome assembly of *A*. *cahirinus* skin and skin wounds. Using a novel computational comparative RNA-Seq approach along with pathway and co-expression analyses, we identify enrichment of regeneration associated genes as well as upregulation of genes directly related to muscle development or function. Our RT-qPCR data reveals induction of the myogenic regulatory factors, as well as upregulation of embryonic myosin, starting between days 14 and 18 post-wounding in *A*. *cahirinus*. In contrast, the myogenic regulatory factors remain downregulated, embryonic myosin is only modestly upregulated, and no new muscle fibers of the panniculus carnosus are generated in *M*. *musculus* wounds. Additionally, we show that *Col6a1*, a key component of the satellite cell niche, is upregulated in *A*. *cahirinus* compared to *M*. *musculus*. Our data also demonstrate that the macrophage profile and inflammatory response is different between species, with *A*. *cahirinus* expressing significantly higher levels of *Il10*. We also demonstrate differential expression of the upstream regulators *Wnt7a*, *Wnt2* and *Wnt6* during wound healing. Our analyses demonstrate that *A*. *cahirinus* is capable of *de novo* skeletal muscle regeneration of the panniculus carnosus following removal of the extracellular matrix. We believe this study represents the first detailed analysis of *de novo* skeletal muscle regeneration observed in an adult mammal.

## Introduction

We have previously shown that, following full-thickness excisional skin wounds or biopsy punches through the pinna of the ear, *A*. *cahirinus* is capable of perfectly regenerating skin, including hairs, sebaceous glands, cartilage, adipose tissue, smooth muscle and even skeletal muscle without fibrosis or scarring [1–4]. Here, we follow up our initial findings with a more comprehensive analysis of gene expression during wound healing in *A*. *cahirinus* and *M*. *musculus* normal (unwounded; day 0) skin and dermal wounds 7 and 14 days after full-thickness excisional wounding. To this end, we have generated a *de novo* transcriptome assembly of *A*. *cahirinus* normal skin and wounds and have performed differential expression analyses using RNA-Seq data. We have also developed a novel computational approach that allows for the direct comparison of changes in gene expression over time between species, termed CORE, that analyzes common orthologous regions between *A*. *cahirinus* and *M*. *musculus*. Co-expression and pathway analyses reveal differences in Wnt signaling, extracellular matrix components, and inflammation pathways, in addition to revealing that *A*. *cahirinus* reactivates the myogenesis pathway, which ultimately leads to *de novo* regeneration of skeletal muscle fibers. This finding was investigated further using RT-qPCR on a time-course of wound healing in both *A*. *cahirinus* and *M*. *musculus* wounds. These results demonstrate that *A*. *cahirinus* is capable of *de novo* skeletal muscle regeneration of the panniculus carnosus (PC) of the skin. We believe this to be the first detailed study of *de novo* skeletal muscle regeneration in an adult mammal.

Skeletal muscle in mammals is one of the few tissues that can repeatedly repair itself throughout life and we have recently described the enhanced regenerative abilities of *A*. *cahirinus* to regenerate skeletal muscle of the tibialis anterior following myotoxin-induced injury [4]. Following injury, muscle regeneration occurs in two major interdependent phases, degeneration followed by regeneration [5]. During the degeneration phase, an initial inflammatory response to necrotic cell death recruits neutrophils and macrophages to the damaged tissue [6]. During this phase, M1, pro-inflammatory macrophages and neutrophils work to remove cell debris. Following the inflammatory phase of degeneration, an increase in *Il10* transitions the macrophage population to a more M2 healing population [7] and the resident muscle stem cells, termed satellite cells, become active, generating a population of proliferating myoblasts which then fuse to form myotubes. As proliferation of satellite cells occurs, they begin to express the myogenic regulatory factors (MRFs) in a temporally induced fashion. Myogenic factor 5 (*Myf5*) and *MyoD* are the first myogenic regulatory factors to be expressed and commit cells down the myogenic program. This is followed shortly thereafter by the expression of the terminal differentiation genes myogenic regulatory factor 4 (*Mrf4*) and myogenin (*Myog*), leading to the fusion of myocytes into myotubes and eventually myofibers. Expression of *Myog* also drives the expression of the embryonic myosin, myosin heavy chain 3 (*Myh3*), in developing and regenerating myofibers. These new fibers then undergo remodeling to mature muscle, resulting in the functional repair of the damaged tissue. (reviewed in [6] (ref)). These new fibers then undergo remodeling to mature muscle, resulting in the functional repair of the damaged tissue [6].

However, in order for skeletal muscle to regenerate, it is an absolute requirement that the existing extracellular matrix (ECM) template be intact following injury [8]. A traumatic injury where skeletal muscle is either removed or destroyed is referred to as a volumetric muscle loss (VML) injury. Following a VML injury, the biomechanical signals of the existing ECM template, basement membrane and connective tissue of the muscle fibers are absent, leading to failed repair and deposition of scar tissue [9]. There is currently no effective treatment to restore muscle volume and function for these types of injuries, and new treatment options are a significant unmet clinical need [10].

Previous work has demonstrated that the inflammatory response to wounding in *A*. *cahirinus* is suppressed compared to that in *M*. *musculus* and that the macrophage profile within *A*. *cahirinus* wounds is quite different [1, 2]. Additionally, we have shown that *A*. *cahirinus* wound dermis appears to be devoid of F4/80 positive macrophages, although these cells are clearly visible at the wound periphery and in the underlying connective tissue fascia [2]. F4/80 has previously been thought to be a pan marker of macrophages [11]; however, Simkin et al. have recently shown that F4/80 +ve macrophages present in wounded ear tissue in *A*. *cahirinus* are not cd206 +ve, a marker of the M2 phenotype, and are likely of the M1, pro-inflammatory phenotype [12]. Here, we expand on these initial reports and show that the *A*. *cahirinus* wound dermis is surprisingly nearly devoid of macropahages in early wounds, although they do appear in later day wounds.

## Results

### Sequencing metrics

Sequencing of both *A*. *cahirinus* and *M*. *musculus* resulted in approximately 42 million read pairs per sample and over 1 billion read pairs total (S1 Table). Trimmed samples contained approximately 29 million reads each with a total of approximately 717 million (S2 Table). Approximately 66% of read pair sequences overlapped and were merged. Quality control identified one *M*. *musculus* sample that exhibited poor and unusual quality metrics in FASTQC [13] and was consequently removed from further analysis.

### *De novo* transcriptome assembly

Assembly of the *A*. *cahirinus* transcriptome (GEO GSE113081) resulted in 283,780 transcript assemblies representing 220,101 Trinity genes with an N50 of 1,794, indicating that at least 50% of the assembled nucleotides are in transcripts at least 1,794 bases long (S3 Table). As a better measure of assembly quality, the ExN50, which represents the N50 for the highest expressed genes accounting for x% of the total normalized expression, was also calculated (S1 Fig) [14]. The maximum N50 in the ExN50 data set was computed to be at E90 (transcripts representing 90% of the normalized expression data), which had an N50 of 2,964 calculated from 29,216 transcripts.

### Functional annotation and ortholog identification

Functional annotation of the *A*. *cahirinus* transcriptome resulted in 128,567 putative transcripts with NCBI BLAST [15] hits to the UniRef90 [16] and/or SwissProt [17] databases using either BLASTX or the predicted TransDecoder [14] proteins and BLASTP (GEO GSE113081). The top taxa with over 500 hits were all mammals with *Mus musculus* containing the most high-scoring segment pairs (S1 Fig). There were 50,982 transcripts with eggnog annotations [18] representing ortholog groups at predefined taxonomic levels, comprising 3,571 unique annotations.

### Expression, co-expression and pathway analyses

**Fig 1:**
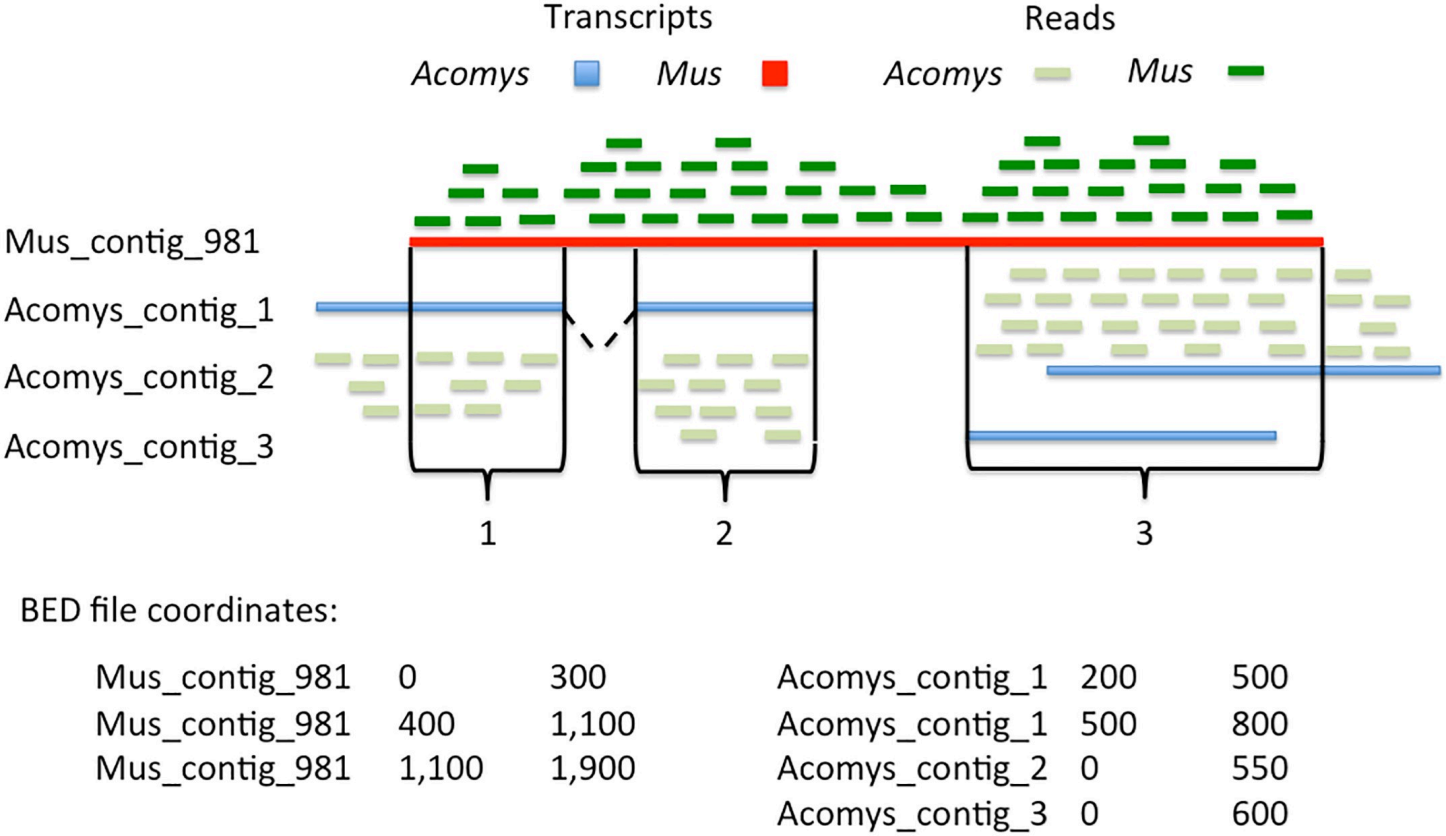
BED file generation and filtering using COREs between transcripts. Putative orthologs identified using a reciprocal best-hit approach are associated via HSPs. HSPs between orthologs are isolated from all hits for reciprocated isoforms allowing the generation of BED files in which coordinates correspond to their species-specific coordinates from the HSP. Multiple *de novo* isoforms from *A*. *cahirinus* are allowed to hit a single *M*. *musculus* transcript. *M*. *musculus* BAM files are filtered using the *M*. *musculus*-specific coordinates, and *A*. *cahirinus* BAM files are similarly filtered.

We identified 21,663 orthologs between *A*. *cahirinus* and *M*. *musculus*, which corresponded to 153,029 Trinity contigs. Read count matrices were generated and differential expression (DE) analyses were performed for *Acomys per se*, *Mus per se* and a combined *Acomys-Mus* analysis (see Methods, Fig 1, S4 Table). Differentially expressed genes from the combined analysis were further examined using Ingenuity Pathway Analysis (IPA) to identify enriched pathways and regeneration-associated genes.

From IPA’s core analysis [19], 218 differentially expressed ‘regeneration’ genes, as defined by IPA, were detected in the combined 7–0 contrast (Day 7 vs Day 0) (S1 Data) and 162 in the 14–0 contrast (S2 Data). We also collected lists of top regulators with their effects. The combined 7–0 contrast contained 51 regulatory pathways such as fatty acid metabolism, cell-cell contact/adhesion, cell proliferation and cell movement (S3 Data). In the combined contrast 14–0, there were numerous muscle related regulatory pathways associated with skeletal muscle contractility, development, damage, cell movement and disorders among its 31 regulator effects (S4 Data).

The combined analysis count matrix was further used to perform a co-expression analysis. The co-expression network generated by petal [20] was filtered at a correlation threshold of 0.914 resulting in a scale-free and small-world network (S2 Fig, S5 Table)[21].

**Fig 2:**
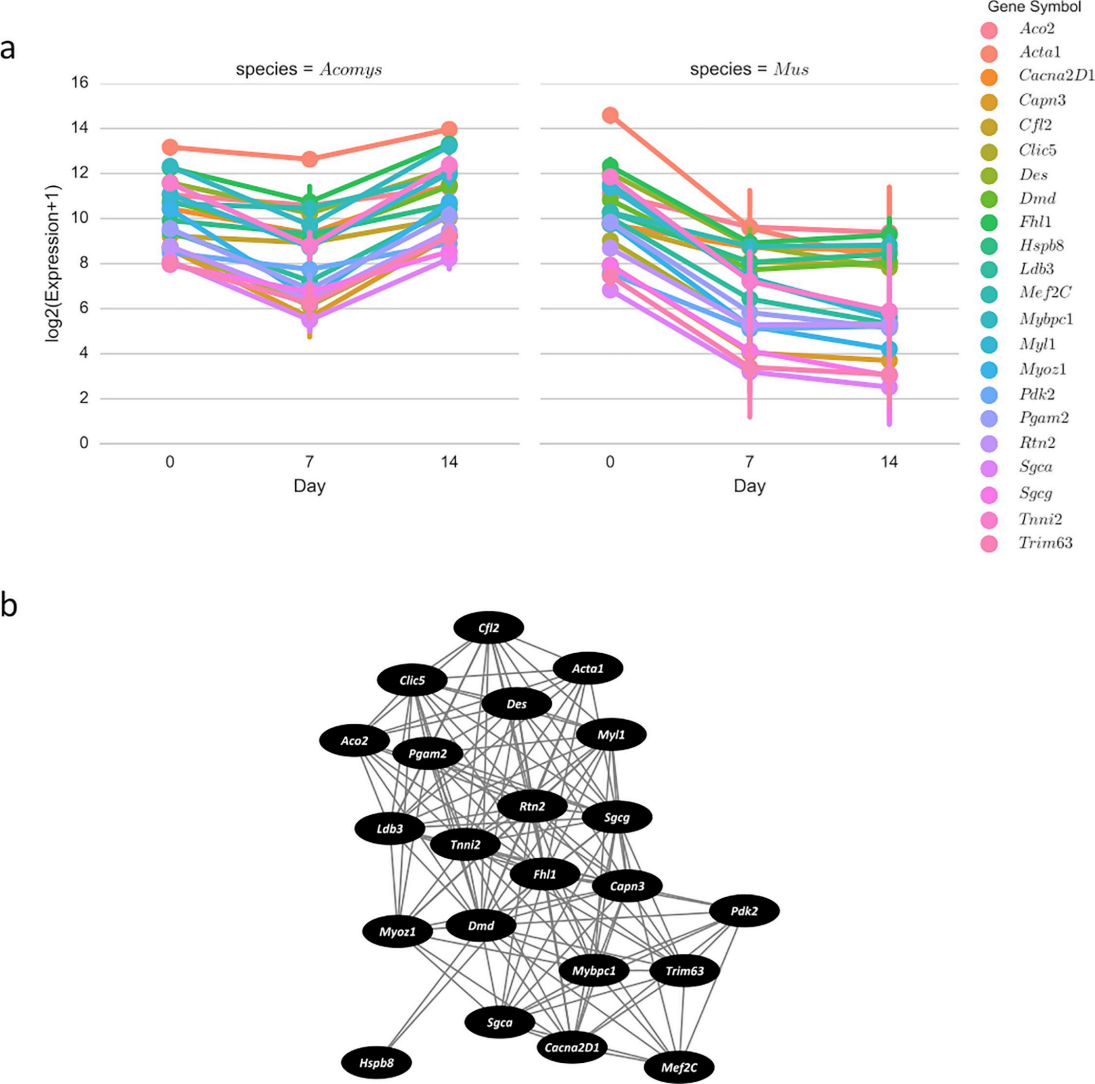
*Dmd* demonstrates co-expression with numerous muscle-related genes. a) Factor plot showing log_2_(RNA-Seq expression + 1) across samples with bars representing 95% confidence intervals. All co-expressed genes are significantly differentially expressed between days 0 and 14 in the combined analysis and represent regulators or regulator targets identified by IPA. b) *Dmd* and co-expressed genes in a network where edges represent a correlation of 0.914 or higher.

The list of regulators and targets obtained from IPA were then used to extract genes of interest from the co-expression network. One subnetwork of interest was extracted using the top upstream regulator, *Dmd*, from the combined 14–0 contrast (Fig 2). *Dmd* codes for dystrophin and is associated with muscular dystrophy when mutated [22]. Essentially all of *Dmd*’s co-expressed genes are associated with some aspect of muscle tissue, e.g. myogenic differentiation and muscle development. As such, these may share some direct or indirect interactions driving regeneration or development of muscle tissue within the excised wound.

### *A*. *cahirinus* upregulates MRFs following full-thickness excisional wounding

In addition to our IPA and co-expression analyses, our RNA-Seq results also demonstrated that 32 of the top 50 genes upregulated in *A*. *cahirinus* day 14 dermal wounds are directly related to muscle development and/or function. To validate these results and to better understand the series of events surrounding the initiation of skeletal muscle regeneration in *A*. *cahirinus*, we examined expression levels of myogenic regulatory factors (MRFs) by RT-qPCR in an expanded time-course of wound healing in both *A*. *cahirinus* and *M*. *musculus*.

**Fig 3:**
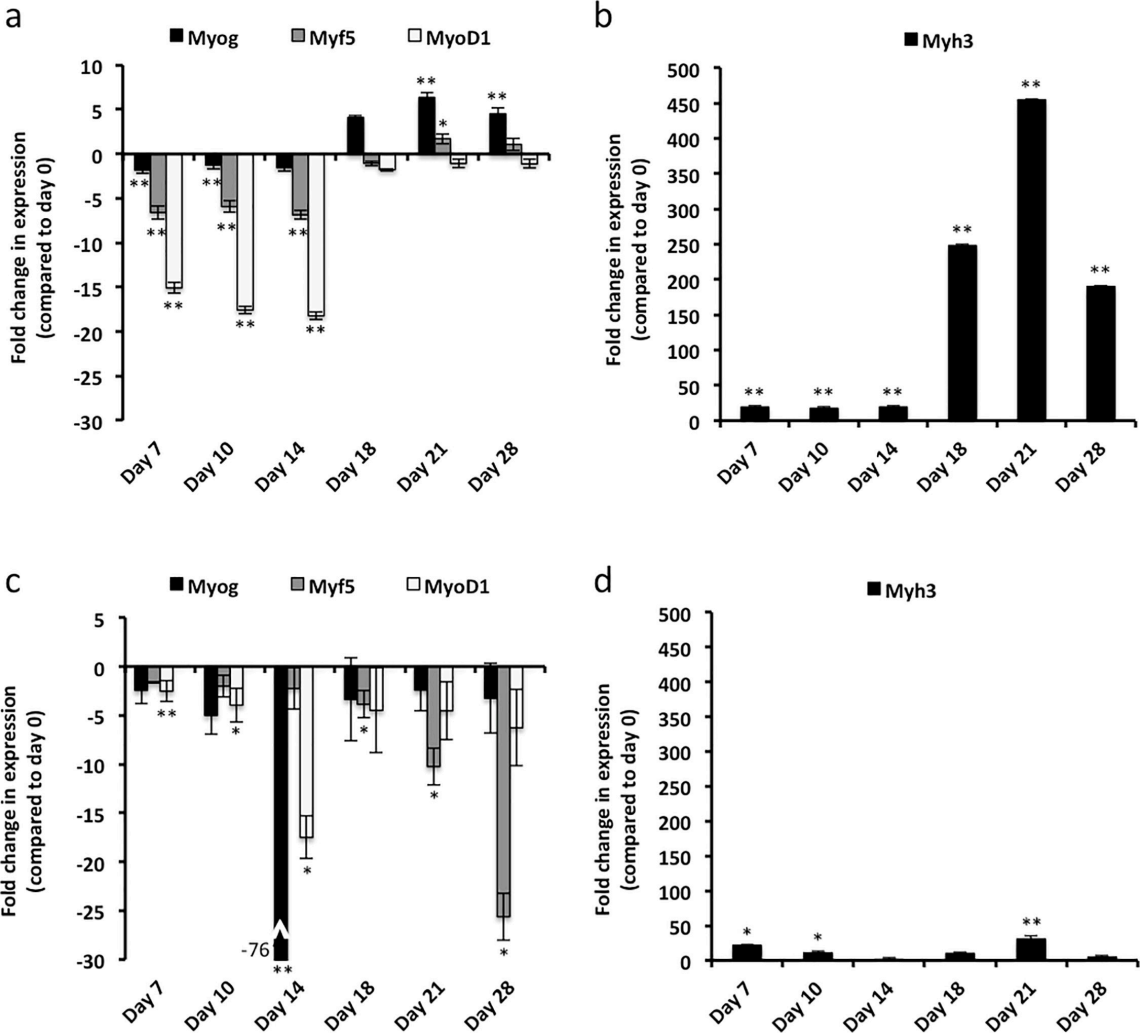
Myogenic regulatory factors and embryonic myosin are upregulated in *A*. *cahirinus* following excisional wounding. RT-qPCR analyses were performed for normal skin and day 7, 10, 14, 18, 21 and 28 wounds (n = 3). (**a**) *A*. *cahirinus* myogenic regulatory factors *Myog*, *Myf5*, and *MyoD1*. (**b**) *A*. *cahirinus* embryonic myosin *Myh3*. (**c)** *M*. *musculus* myogenic regulatory factors *Myog*, *Myf5*, and *MyoD1*. (**d**) *M*. *musculus* embryonic myosin *Myh3*. All values are expressed as fold change compared to day 0 normal skin. Error bars are calculated using standard error propagation. P-values are as follows: * ≤ 0.05; ** ≤ 0.01.

Expression of the MRFs in *A*. *cahirinus* follows the same basic pattern over time. The levels of *Myog*, *Myf5* and *MyoD* mRNA are reduced in day 7–14 samples, as the PC is completely removed in full-thickness excisional wounds. Expression of the MRFs is upregulated between days 14 and 18, and remains high through day 28 (Fig 3A). Expression of the embryonic myosin *Myh3* is moderately upregulated in day 7–14 wounds. Following upregulation of the MRFs, *Myh3* is induced to very high levels in day 18, 21 and 28 wounds, as compared to normal skin (Fig 3B).

In contrast to this, neither the MRFs nor embryonic myosin are induced to a high degree in *M*. *musculus* at any of the examined time points (Fig 3C and 3D). Expression of *Myog*, *Myf5* and *MyoD1* is unchanged, or downregulated, relative to unwounded skin at all time points assayed (Fig 3C). Expression of *Myh3* is unchanged in day 14, 18 and 28 wounds and upregulated in day 7, 10, and 21 wounds; however the increase is at least an order of magnitude lower than that observed in *A*. *cahirinus* (Fig 3D).

### Newly formed muscle fibers in *A*. *cahirinus* express embryonic myosin

**Fig 4:**
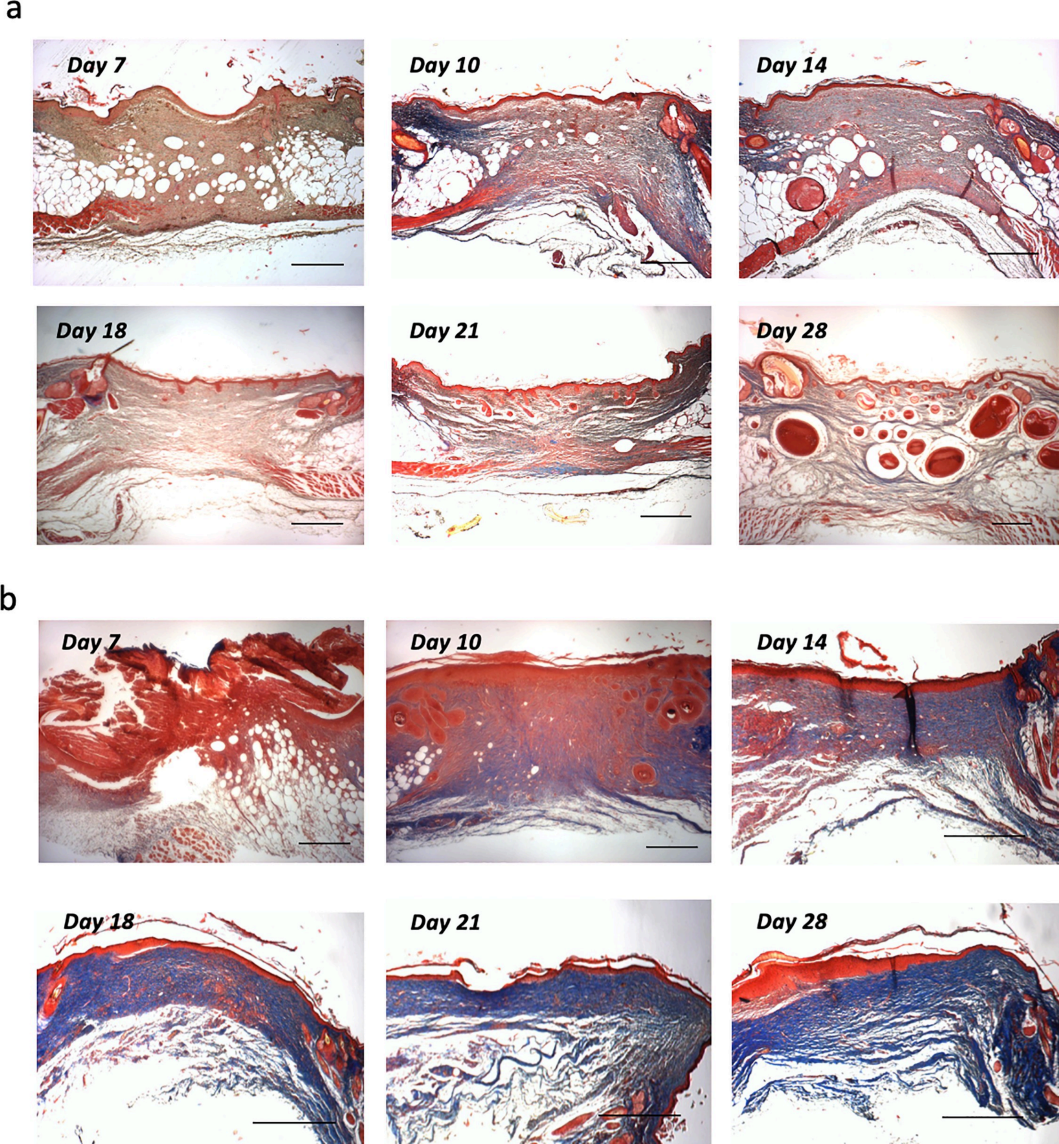
Histological analysis of wound healing reveals *de novo* regeneration of muscle fibers in *A*. *cahirinus*. Masson Trichrome staining was performed for paraffin wax embedded sections for day 7, 10, 14, 18, 21 and 28 wounds for (a) *A cahirinus* and (b) *M*. *musculus*. Scale bars 200 μm.

**Fig 5:**
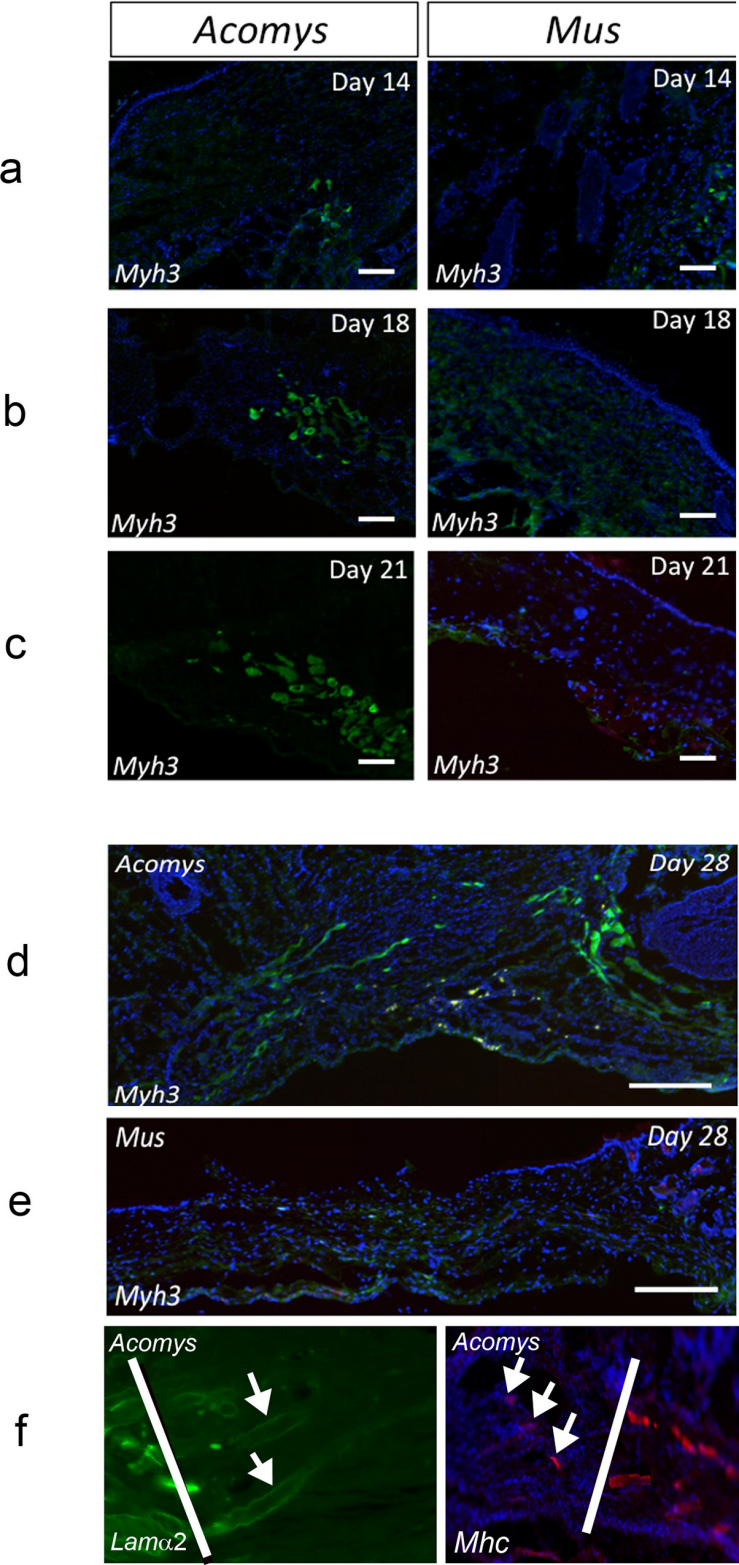
Embryonic myosin *Myh3* staining indicates newly formed muscle fibers in *Acomys* but not *Mus* wounds. Immunofluorescence was performed on fresh, unfixed cryosectioned tissues for *Myh3* on (**a**) day14, (**b**) day 18 and (**c**) day 21 wound sections. Day 28 sections for *Myh3* spanning entire wound for (**d**) *A*. *cahirinus* and (**e**) *M*. *musculus*. (**f**) Day 28 sections white lines mark the wound edge. Left panel, section stained with laminin a2 showing positive fibers in the regenerated part of the wound (right of the white line). Right panel, section stained with myosin heavy chain showing patchy staining in the normal pc (right of the white line) and in the wound (left of the white line). Blue–Hoechst. Scale bars 100 μm.

Histological examination of wound healing using Mason’s Trichrome reveals filamentous tissue, originating from the PC at the wound margin, forming between days 7 and 10 after wounding in *A*. *cahirinus* (Fig 4A). In contrast, *M*. *musculus* wounds stain intensely blue, indicating a dense collagenous matrix (Fig 4B). In *A*. *cahirinus* this tissue becomes better defined and resembles newly formed muscle fibers between day 18 and 28, while in *M*. *musculus* the entirety of the wound is a dense collagenous matrix (Fig 4). To ascertain if this new filamentous tissue in the *A*. *cahirinus* wound bed is indeed regenerating skeletal muscle, we assayed wounds by immunohistochemistry (IHC) using an antibody to embryonic myosin (*Myh3*). In *A*. *cahirinus*, *Myh3* positive cells are first visible at the wound margin by day 14 but do not yet appear to have migrated into the wound (Fig 5A). By day 18 *Myh3* staining is visible beyond the wound margin and into the wound bed itself. Additionally, there is *Myh3* staining visible in more of the intact PC of the surrounding tissue (Fig 5B). By day 21, new myofibers are clearly visible well into the wound while staining of the intact PC remains near the cut edge (Fig 5C). By day 28, new regenerating fibers are visible across the wound from end to end (Fig 5D). These newly regenerated fibers not only express embryonic myosin but some fibers also express the mature muscle marker laminin a2 (Fig 5F, left panel) and are beginning to show the same patchy expression of myosin heavy chain that the normal panniculus carnosus does (Fig 5F, right panel).

In stark contrast to this, *Myh3* staining is only visible in the remaining intact PC at the wound margin in *M*. *musculus* day 14 wounds (Fig 5A). At later time points in *M*. *musculus* wounds very little *Myh3* staining is visible and no laminin a2 or myosin heavy chain immunoreactivity, suggesting that the PC degenerates near the cut edge of the wound margin, and at no point are new regenerating fibers visible (Fig 4 and Fig 5B–5E).

### Key collagens are differentially expressed during wound healing between *A*. *cahirinus* and *M*. *musculus*

**Fig 6:**
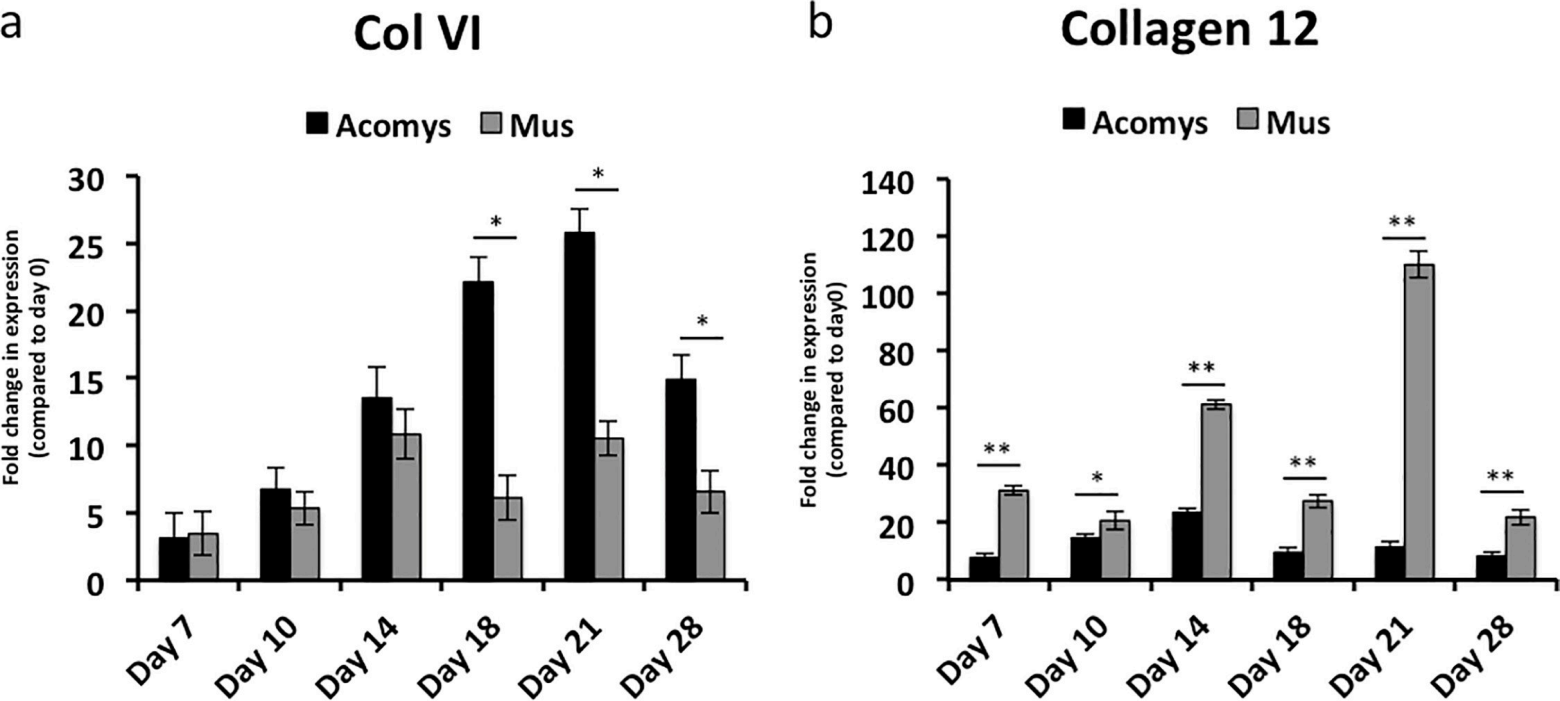
Differential expression of key collagens during wound healing between *A*. *cahirinus* and *M*. *musculus*. RT-qPCR analyses were performed for (**a**) *Col6a1* and (**b**) *Col12a1*. All labels, symbols and calculations are as described in **Fig 3**. Black bars represent *A*. *cahirinus* and grey bars represent *M*. *musculus* data. All fold changes are statistically significant (p-value ≤ 0.05) as compared to each species normal skin day 0 sample. Asterisk over horizontal line indicates statistical significance between species for that time point.

Previous dogma suggested that an existing ECM template was an absolute requirement for skeletal muscle regeneration and that specific components of the stem-cell niche, such as collagen 6a1 (*Col6a1*), are critical to satellite cell function [23]. Therefore, we asked whether *Col6a1* was differentially expressed during regeneration of the PC. *Col6a1* is upregulated in both species at all wound time points assayed and is statistically upregulated in *A*. *cahirinus* compared to *M*. *musculus* in day 18, 21 and 28 wounds (Fig 6A), which coincides with the induction of the MRFs and embryonic myosin (Fig 3A and 3B), consistent with its role in skeletal muscle regeneration.

To further investigate the composition of the ECM of wounds, we next assayed for the expression of collagen 12a1 (*Col12a1*). We have previously shown that *Col12a1* is wound induced in *M*. *musculus* and appears to be a major constituent of wound matrix in day 14 wounds [2]. Here we performed a time course analysis of expression throughout wound healing. While *Col12a1* is statistically upregulated in both species, expression increased to a statistically greater degree in *M*. *musculus* at all time points assayed (Fig 6B).

### Inflammatory response to wounding

We have previously reported that the inflammatory response to wounding in *A*. *cahirinus* is suppressed compared to that in *M*. *musculus* and that the macrophage profile within *A*. *cahirinus* wounds is quite different [1, 2]. Simkin et al. have recently shown that F4/80 +ve macrophages present in wounded ear tissue in *A*. *cahirinus* are not cd206 +ve, a marker of the M2 phenotype, and are likely of the M1, pro-inflammatory phenotype [12]. As M2 macrophages are generally thought to be beneficial, we next asked whether cd206 +ve macrophages were present in dorsal dermal wounds.

**Fig 7:**
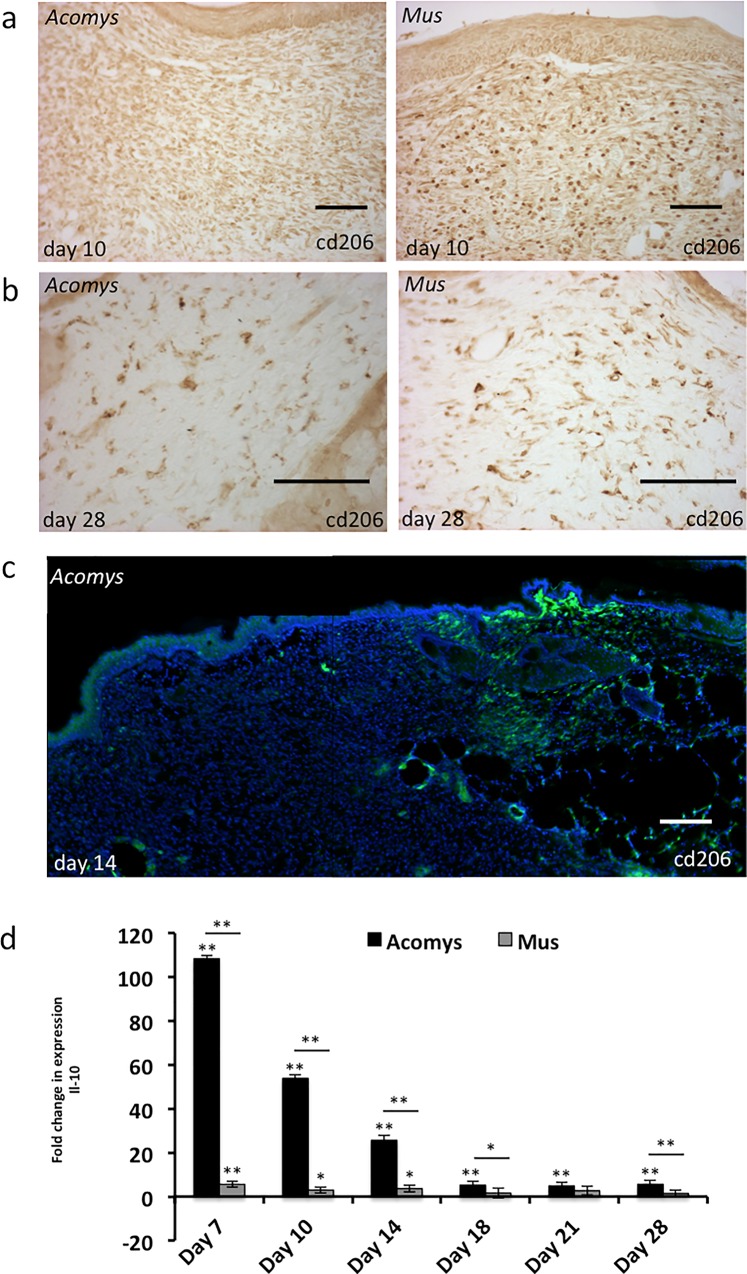
Immune response to wounding. Immunohistochemistry performed with paraffin wax embedded samples for cd206 in (**a**) day 10 and (**b**) day 28 wounds. (**c**) Immunofluorescence was performed for cd206 on fixed cryosections. Scale bars 100 μm. (**d**) RT-qPCR analysis of *Il10*. Asterisks over specific sample indicate significance to that species normal skin day 0 sample, while asterisks over line spanning both species indicates significance between species. Green–cd206; Blue–Hoechst. All other labels, symbols and calculations are as described in **Fig 3**.

Macrophages positive for cd206 staining are present in the wound bed and dermis at all time points assayed in *M*. *musculus* dermal wounds, similar to our previous results for F4/80. However, cd206 +ve macrophages are surprisingly absent in the early *A*. *cahirinus* wound bed, up to day 10 (Fig 7A). By day 14, there are low numbers of cd206 +ve macrophages beginning to appear directly underneath the wound epidermis, despite great numbers being present at the wound margins (Fig 7C). By day 18, cd206 +ve macrophages are present throughout the dermis with essentially the same number and distribution as in *M*. *musculus* and remain the same through day 28 wounds (Fig 7B). We next utilized another widely used pan-macrophage marker, ionized calcium-binding adaptor molecule 1 (*Iba-1*). Immunostaining in *A*. *cahirinus* wounds showed that there are a small number of *Iba-1* +ve cells present just below the wound epidermis in day 7 and 10 wounds, while the dermis remains largely devoid of *Iba-1* macrophages (S3 Fig), similar to our previous observation for F4/80 and cd206 +ve macrophages. By day 14, *Iba-1* +ve macrophages are present similar to cd206 +ve macrophages in both species (S3 Fig).

As the transition of macrophages from M1 to an M2 phenotype is thought to be critical for the progression of myogenic differentiation [7] we next examined the expression of interleukin 10 (*Il10*), an anti-inflammatory cytokine [24–27], that has been shown to transition macrophages to an M2 phenotype [28]. *Il10* expression was assayed by RT-qPCR in a time-course analysis of wound healing in both *A*. *cahirinus* and *M*. *musculus*. *Il10* expression was upregulated to a much greater extent in *A*. *cahirinus* wounds than in *M*. *musculus*, with expression of *Il10* peaking in *A*. *cahirinus* at or before day 7 (~100x) and declining by half at day 10 and half again at day 14, and remaining upregulated ~ 5-fold in day 18–28 wounds (Fig 7D). *Il10* expression in *M*. *musculus* is upregulated as well in day 7, 10 and 14 wounds, although to a much lesser extent than observed in *A*. *cahirinus*, and remains unchanged in day 18–28 wounds (Fig 7D).

### Differential expression of key *Wnts* observed during wound healing between *A*. *cahirinus* and *M*. *musculus*

To gain a better understanding of the upstream regulatory events that precede the observed regeneration in *A*. *cahirinus* we examined the expression profiles of three Wnts implicated in skeletal muscle development (*Wnt6*) [29–31], muscle regeneration [32] and hair follicle neogenesis [33] (*Wnt7a*) and in the fibrotic response (*Wnt2*) [34] by qRT-PCR.

**Fig 8:**
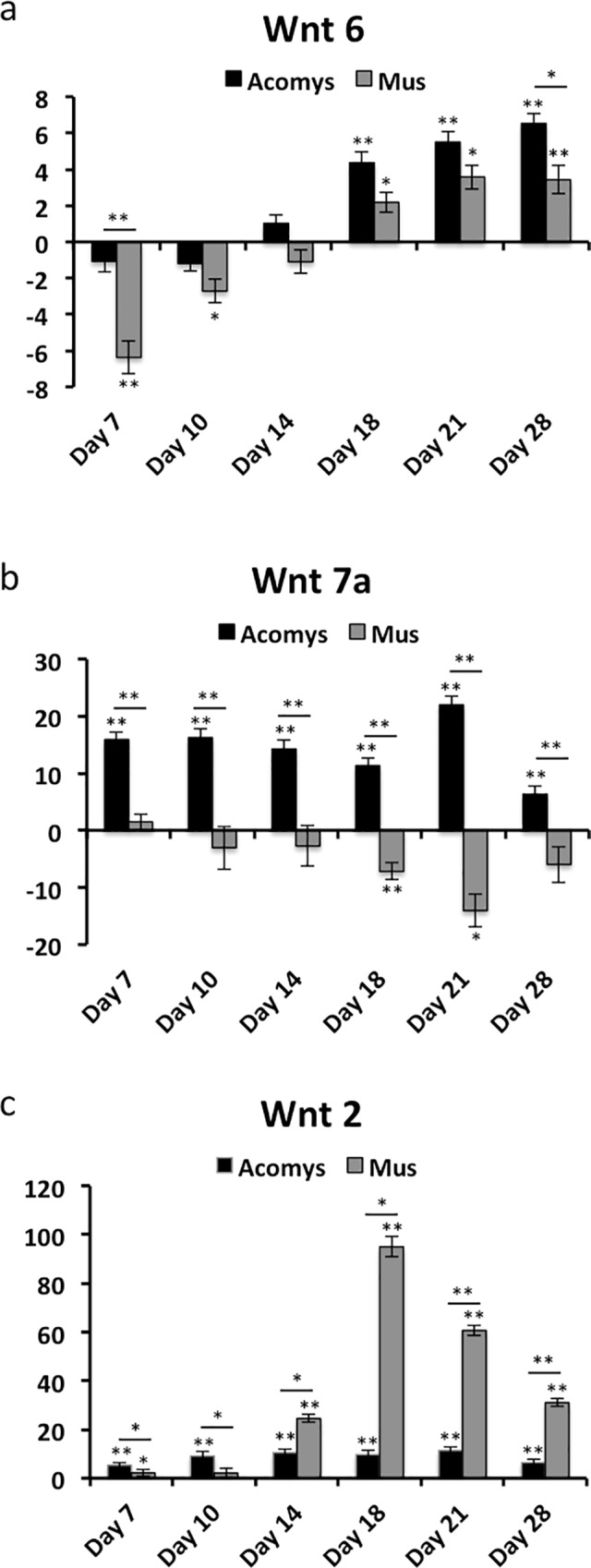
Differential expression of key Wnts during wound healing between *A*. *cahirinus* and *M*. *musculus*. RT-qPCR analyses were performed for (**a**) *Wnt6*, (**b**) *Wnt7a* and (**c**) *Wnt2*. All labels, symbols and calculations are as described in **Fig 7**.

Expression levels of *Wnt6* in *A*. *cahirinus* are essentially unchanged in day 7–14 wounds, while in *M*. *musculus Wnt6* is downregulated in day 7 and 10 and unchanged in day 14 wounds. Expression of *Wnt6* is statistically upregulated in day 18–28 wounds in both *A*. *cahirinus* and *M*. *musculus*. While the trend is for increased expression in *A*. *cahirinus* at all time points assayed compared to *M*. *musculus*, only days 7 and 28 show statistically significant differences in expression between species (Fig 8A). The increased expression of *Wnt6* also coincides with the induction of the MRFs, *Myh3* and *Col6a1* (Fig 3A and 3B) consistent with its role in skeletal muscle development.

*Wnt7a* is significantly upregulated in *A*. *cahirinus* wounds, both compared to normal skin and to *M*. *musculus*, at all time points assayed, while *Wnt7a* expression in *M*. *musculus* is either unchanged or downregulated at all time points (Fig 8B. This is consistent with *Wnt7a’s* role in skeletal muscle regeneration and hair follicle neogenesis [32, 33]. *Wnt2* has been shown to be associated with fibrosis [34] and we therefore asked whether *Wnt2* is differentially expressed during wound healing.

*Wnt2* expression is elevated in *A*. *cahirinus* day 7 and 10 wounds relative to day 0, and this difference is greater than the difference between *M*. *musculus* day 7 and 10 relative to day 0. *Wnt2* expression remains modestly elevated in *A*. *cahirinus* in day 14–28 wounds, compared to normal skin. In *M*. *musculus* however, expression of *Wnt2* is induced to high degree starting at day 14, peaking at day 18 and slowly decreasing in days 21 and 28 wounds (Fig 8C). The high level of expression coincides with the tissue-remodeling phase, and may help explain why the *M*. *musculus* wound is remodeled as scar tissue. This is consistent with the observed role of *Wnt2* in regulating genes associated with fibrosis [34].

## Discussion

To assess transcriptome completeness, we used BUSCO to identify near-universal single-copy orthologs in vertebrata (S1 Fig). Compared to previous *A*. *cahirinus* transcriptome assemblies, our assembly represents a substantial increase in the identification of complete and single-copy orthologs. Other assemblies contained many more duplicated transcripts with one assembly containing over 2.2 million transcripts [35]. While that same assembly had fewer missing single-copy orthologs, it also represented 15 different organ types. In a slightly more comparable assembly based upon a time series analysis of wounded ear tissue, single-copy ortholog fragmentation was high along with increased duplication and missing transcripts [36]. Differences are likely due to the lower coverage with shorter reads and tissue-specific expression. As another metric indicative of transcriptome assembly quality, we provide the ExN50 quality metric, which indicates that 90% of the total normalized expression results from 29,087 transcripts as opposed to expression being dispersed broadly over many fragmented transcripts. The ExN50 plots the most highly expressed transcripts representing x% of the total normalized expression data (S1 Fig). The E90N50 of our transcriptome assembly has an N50 of 2,932 and is composed of 29,087 transcripts representing 90% of the total normalized expression. We also provide a descriptive annotation and statistics of our assembly (GEO GSE113081, S3 Table). Together these metrics indicate a superior transcriptome assembly representative of regeneration-associated expression in *A*. *cahirinus* skin.

There are numerous difficulties associated with comparing differential expression between two different species such as different quality references, structural differences between genomes or transcriptomes, different basal expression levels, identification of orthologs, and the lack of methods to make these comparisons. Of the attempts made to date, some procedures perform independent analyses of both species and attempt to reconcile differences based upon set comparisons of differentially expressed genes [37]. However, this approach is sensitive to the choice of significance cutoff, is ineffective for experiments with low power and may suffer technical biases if different treatments are compared to the same control [38]. A few studies use species for which genomes and annotations are readily available and limit the analysis to conserved regions [39–41]. This makes reconciling differences between species a matter of comparing the genomes and using constitutively expressed exons common to the species under consideration [39]. With the advent of *de novo* transcriptome assemblies, the opportunities for cross-species comparisons have drastically increased, but the methods available to make these comparisons are still lacking.

Here, we identified Common Orthologous REgions (COREs) between species transcriptomes so that direct comparisons of gene expression may be made (Fig 1) as well as perform an individualized analysis for each species. The individualized analyses will be referred to as *Mus per se* and *Acomys per se*. The cross-species analysis will be referred to as the combined analysis. The *per se* models, which represent the more traditional approach, allow us to analyze differential expression within a single species using pairwise contrasts. In this scenario, comparisons between species are limited to comparing log-fold changes or differentially expressed genes. The combined model is limited to homologous regions between orthologs but allows for a direct comparison between species, which is especially important considering the basal expression levels differ considerably for some genes between the two species. In these cases, comparing log-fold changes or differential expression between species could be misleading. The contrast for the combined model also differs in that it is a time-series contrast that assesses the differences between species pairwise contrasts.

In the combined analysis, the position of species in the multi-dimensional scaling plot makes biological sense as the *A*. *cahirinus* day 14 wounds are observably more similar to day 0 as the tissue has progressed in the regeneration of the skin (S1 Fig). Conversely, the tissue in *M*. *musculus* day 14 has not regenerated and more closely resembles the day 7 wounds. Expression patterns match biological expectations and previous microarray data [1].

Generating gene co-expression networks to detect multi-species gene module conservation is an increasingly popular tool for RNA-Seq studies [20, 42, 43]. Co-expression networks are just one of many biological systems that demonstrate network dynamics and have been extensively observed [44–47]. These networks exhibit small-world properties, which are characterized by high-connectivity among subnetworks resulting in relatively short paths to move between any two nodes [48], and scale-free properties, which are characterized by many nodes with few connections and few nodes with many connections [49]. For a network to exhibit the small-world property, the average path length between nodes is expected to be shorter than a random graph, and the average cluster coefficient is expected to be larger than random [48]. Similarly, the network is expected to be scale-free, which requires that the node degree distribution follow a power-law distribution. Here, we use the R package petal, which uses these features to dynamically determine a correlation threshold for network generation [21]. While petal uses least squares to fit the log-transformed degree distribution to check for a power-law distribution, this practice is not statistically robust [21](S4 Fig), and as such, we further validated the power-law distribution using bootstrapped maximum likelihood estimation [21](S5 Fig). This approach also confirmed that the network was scale-free.

We also performed a pathway analysis using IPA [19]. This approach differs from a co-expression analysis in that differentially expressed genes from our study are used in conjunction with curated data from other studies providing additional information from previous co-expression, interactions (gene-gene, protein-protein, gene-protein, etc.), known functionality (wound healing, regeneration, muscle contraction, etc.) and more. We found that, based upon our combined analysis, there were numerous regulators associated with cell-cell contact, cell proliferation, movement and recruitment up-regulated in the day 7–0 contrast. Whereas the day 14–0 contrast regulators included those associated with muscle development and contraction, fibroblast movement, myopathy, and muscle damage.

Genes of interest were extracted from the co-expression network using the lists of regulators and their downstream targets from IPA’s regulatory effects analysis to generate factor plots depicting expression patterns of regulatory genes and targets that are co-expressed and differentially expressed. The *Dmd* subnetwork indicates that numerous muscle-related genes exhibit correlated expression patterns following wounding (Fig 2). Expression levels of these co-expressed genes are lower in day 7 wounds, in both *A*. *cahirinus* and *M*. *musculus*. This makes sense biologically as all muscle tissue is removed following excisional wounding. By day 14, expression increases in *A*. *cahirinus* when skeletal muscle starts to form, while expression stays decreased in *M*. *musculus* as no muscle tissue is regenerated. Histological analysis of wounds from later time-points suggests that muscle regeneration becomes more robust after day 14.

It was previously believed that muscle regeneration in mammals could only occur in the presence of an existing ECM template [8]. However, based on our current study, *A*. *cahirinus* appears capable of *de novo* regeneration of muscle of the PC following removal of the instructive ECM template. As muscle regeneration shares many of the same molecular programs as myogenesis during fetal development [6], we first assayed expression of the MRFs and embryonic myosin. In both embryonic and adult myogenesis the MRFs are expressed in a temporally induced fashion, with *Myf5* and *MyoD1* expression occurring first, followed by expression of *Myog*, which induces the expression of the embryonic myosin *Myh3*. Following tissue excision, the MRFs are downregulated in both species; however, after day 14 the MRFs return to expression levels of normal tissue and remain at normal or elevated levels in *A*. *cahirinus*, indicating that myogenesis has begun. Curiously, expression of *Myh3* in *A*. *cahirinus* is upregulated in early wounds, before upregulation of the MRFs is detectable by RT-qPCR and may represent repair of the injured fibers at the wound margins. However, concurrent with upregulation of the MRFs, *Myh3* is induced to a high degree in *A*. *cahirinus* wounds indicating a robust regeneration of muscle fibers is occurring. As our RT-qPCR data represent expression changes that are occurring in the whole wound tissue, we next sought to verify that new muscle fibers are indeed being formed by assaying wounds by IHC using an antibody against *Myh3*. In *A*. *cahirinus* day 14 wounds, *Myh3* staining is visible in the intact PC at the wound margin but does not appear to have migrated into the wound bed. Additionally, *myh3* staining appears to be localized to a region near the severed fibers at the wound margin and is not visible further into the intact PC, suggesting a rather localized response. By day 18, *myh3* positive fibers are visible beyond the wound margin and into the wound bed. Additionally, the morphology of these fibers suggests that the PC is degenerating near the wound margin with small isolated cross-sectional fibers visible similar to what is observed following myotoxin injury of skeletal muscle [4, 50]. By day 21, *myh3* positive fibers are visible well into the wound tissue, and are completely spanning the wound by day 28, suggesting the *de novo* formation of functional muscle fibers of the PC which was further supported by the appearance of laminin a2 and myosin heavy chain immunoreactivity.

In stark contrast to *A*. *cahirinus*, the MRFs in *M*. *musculus* wounds remain downregulated at all time points assayed. Interestingly, there appears to be a large decrease in expression at day 14 for *Myog* and *MyoD1*. This expression pattern is confirmed in our RNA-Seq data, which was performed on independent animals. Considering that *MyoD1* promotes expression of *Myog*, which drives expression of *Myh3*, it is interesting to note that at this time point *myh3* is at its lowest level. While expression of *Myh3* is upregulated in *M*. *musculus* early wounds, no *Myh3* positive fibers are visible in the wound and may just represent either the repair occurring at the wound margins or an unproductive attempt at regeneration.

*Col6* is a major component of skeletal muscle ECM and has been shown to be critical for proper function of satellite cells [23]. Our data indicate that *Col6a1* is significantly upregulated in *A*. *cahirinus* during the time that new muscle fibers are observed in the wound tissue, suggesting a role in muscle ECM formation [2].

During the regeneration phase of muscle repair, when new muscle fibers are being formed, there is also scar tissue being deposited. In small injuries, this scar tissue acts to bridge the gap between muscle fibers and provides a means of propagating force along the remaining muscle, thus restoring function. However, in large injuries, such as those observed in VML injuries, the formation of scar tissue forms a dense cap of fibrotic tissue that blocks the regeneration of new muscle fibers and results in reduced function of the repaired muscle [9]. We have previously shown that *Col12*, a small cross-linking collagen, is wound induced in *M*. *musculus* in skin wounds [1] and in the TA following myotoxin induced injury [4]. The resulting dense fibrotic tissue may be acting to block regenerating muscle fibers from migrating into the wound tissue. This is particularly interesting as *Wnt2* is also significantly upregulated in *M*. *musculus* compared to *A*. *cahirinus* during the period when muscle regeneration and tissue remodeling would be occurring. Previous work by Bayle et al. have shown that *Wnt2* is over-expressed in the tight-skinned mouse (Tsk), which is used as a model of systemic sclerosis, a connective tissue disorder associated with increased cutaneous fibrosis [34]. The authors suggest that over-expression of *Wnt2* contributes to the aberrant matrix remodeling associated with the Tsk mouse phenotype.

*Wnt6* has been shown to be involved in muscle development [29–31] and our RT-qPCR data show an expression pattern similar to that of the MRFs and embryonic myosin in *A*. *cahirinus*, consistent with a role in myogenesis. Interestingly, the expression of *Wnt6* is also significantly upregulated in *M*. *musculus*. Taken together with the expression patterns for *Wnt2* and *Col12*, this suggests that *Wnt6* may be providing the signal for myogenesis in *M*. *musculus*, but fails in regeneration due to the strong fibrotic response of *Wnt2* signaling.

We were also interested in the expression of *Wnt7a*, another Wnt that has been shown to be involved in skeletal muscle regeneration [32] and hair follicle neogenesis [33]. *Wnt7a* is significantly upregulated in *A*. *cahirinus*, while it is unchanged or downregulated in *M*. *musculus*. These expression patterns are consistent with the role of *Wnt7a* since *A*. *cahirinus* regenerates both skeletal muscle and hair follicles and *M*. *musculus* regenerates neither.

Macrophages play a critical role in wound healing and in muscle regeneration. Early in wound healing, pro-inflammatory phagocytic M1 macrophages remove necrotic tissue and cell debris, while in later wounds macrophages transition to an M2 phenotype. This transition to M2 is facilitated by upregulation of *Il10* and has been shown to be critical in proper muscle regeneration [7]. Our expression data shows that *Il10* is upregulated to a high degree in A. cahirinus and is statistically upregulated compared to *M*. *musculus*. While RT-qPCR data shows a very high fold change in expression of *Il10*, the RNA-seq shows only a moderate increase. This discrepancy can be explained by the fact that *Il10* expression in day 0 samples is quite low in both our RNA-Seq (S6 Fig) and RT-qPCR datasets, making it difficult to accurately predict fold change. Combined with our data for M2, cd206 positive macrophages, this data suggests that the increased levels of *Il10* in *A*. *cahirinus* may provide an environment for more M2 macrophages in the wound and may promote regeneration over fibrosis. However, it is still not clear whether the early dermis has a true absence of macrophages or if there is an unobserved subtype present.

Our study demonstrates that *A*. *cahirinus* is capable of *de novo* regeneration of skeletal muscle of the PC following full-thickness excisional wounding. We believe this to be the first detailed analysis of *de novo* skeletal muscle regeneration occurring in an adult mammal. Additionally we have presented data suggesting a role of Wnt signaling in the initiation of regeneration. The differential expression patterns of Wnts between *A*. *cahirinus* and *M*. *musculus* demonstrate the importance of these upstream regulators and suggest Wnts as potential targets for manipulation to favor muscle regeneration over fibrosis in VML injuries in future studies.

## Methods

### Animals

Animal protocols were approved by the Institutional Animal Care and Use Committee (IACUC) at the University of Florida (Protocol numbers 201207707 for *A*. *cahirinus* studies and 201203505 for *M*. *musculus* studies). All animals were housed and maintained by Animal Care Services at the University of Florida and all experiments were performed following the guidelines defined in the *Guide for the Care and Use of Laboratory Animals of the National Institutes of Health*. *Acomys cahirinus* were obtained from an in-house breeding colony and CD-1 mice were obtained from Charles River Laboratories. All surgeries were performed under isoflurane inhalation anesthesia and all efforts were made to minimize suffering. All animals were aged between 6 weeks and 6 months of age. One 8-mm full-thickness biopsy punch was excised from the shaved dorsal skin and immediately placed in RNAlater Stabilization Solution (Invitrogen AM7020) and stored at 4°C for 24hrs then stored at -80°C. Normal dorsal skin was used as a day 0 control. For our initial analysis of gene expression, and transcriptome assembly, we excised wounds from day 0, 7, and 14 wounds. These time-points were chosen based on the major observable events during wound healing (inflammation and granulation (day 7) and tissue remodeling (day 14)) and to complement data from our previous analysis of gene expression using microarrays ^1^. In order to further explore the observed increase in myogenic reated genes, we performed RT-PCR analysis on our initial time-points, plus the addition of day 10, 18, 21 and 28 wounds. Tissues were excised, excluding the normal surrounding tissue for RNA-Seq and RT-PCR and including the surrounding tissue of the wound margins for histology (n = 4 for each time point for each species for RNA-Seq; n = 3 for each time point for each species for RT-qPCR).

### RNA extraction

Tissue samples were removed from -80°C, thawed at 4°C, removed from RNAlater and washed in nuclease-free water to remove RNALater crystals. Tissue was homogenized using a 1600 MiniG Tissue Homogenizer and Cell Lyser (SPEXSamplePrep) with 5/32” stainless-steel grinding balls in TRIzol (Ambion) in 2ml screw-cap vials at 1500 RPM for 3 minutes. After homogenization, TRIZol protocol was followed with modifications. Briefly; samples were transferred to Phase Lock Gel Heavy 2mL tubes (QuantaBio; VWR 10847–802). After aqueous phase removed to new tube, 1 volume of 70% ethanol was added and samples mixed. At this point samples were processed according to the RNeasy Mini Kit (Qiagen 74104) or the PureLink RNA Mini Kit (Invitrogen 12183018A) according to manufactures protocol, starting from addition of sample to kit column tubes. After elution, samples were quantified and RNA integrity number (RIN) determined using a 4200 TapeStation (Agilent) (All RINs for RNA-Seq > 7 (avg 7.7), and > 6 for RT-PCR).

### RNA-Seq Library construction and illumina sequencing

Illumina ribo-depleted, 2 x 150 bp paired-end libraries were constructed using total RNA from 24 samples composed of 4 replicates per time point (day 0, 7 and 14) per species (*A*. *cahirinus* and *M*. *musculus*). Additionally, a duplex-specific nuclease (DSN) library was constructed using RNA from all 12 *A*. *cahirinus* samples. All 25 libraries were sequenced using an Illumina NextSeq500. Library construction and sequencing was performed by Cofactor Genomics (St. Louis, Missouri).

### RNA processing

Raw reads were quality assessed pre- and post-filtering using FastQC [fastqc/0.11.4] [13] and trimmed using Trimmomatic [trimmomatic/0.32, ILLUMINACLIP:TruSeq3-PE-2.fa:2:30:10 HEADCROP:10 LEADING:3 TRAILING:3 SLIDINGWINDOW:4:15 MINLEN:40] [51]. Trimmed reads were merged using SeqPrep [seqprep/1.1, -L 40 -n 0.95 -o 25] [52] resulting in both unmerged paired-end reads and merged reads (average ~250-bp in length).

### *De novo* assembly and annotation of *A*. *cahirinus* transcriptome

*A*. *cahirinus* merged and paired-end RNA-Seq reads from all three time-points, plus the DSN library, were assembled using Trinity [trinity/2.0.4,—SS_lib_type FR—min_contig_length 500] [53], with *in silico* normalization at 50X coverage to remove systemic variation. The resulting assembly was functionally annotated using Trinotate [trinotate/2.0.1] [14], which utilizes a variety of homology searches to publically available data to identify homologous sequences (SwissProt/UniRef90 [16, 17]), protein domains (HMMER/PFAM) [54, 55], rRNA (RNAMMER) [56], protein signal peptides and transmembrane domains (signalp/tmHMM) [57–59]. The annotation report only includes hits meeting a minimum E-value cutoff of 1E-5.

### Ortholog identification

Orthologs were identified between *A*. *cahirinus* and *M*. *musculus* using a reciprocal best-hit BLAST approach between *A*. *cahirinus* transcripts and *M*. *musculus* RefSeq [60] sequences at E-value ≤ 1E-5 using WU-BLAST BLASTN [61] where multiple *A*. *cahirinus* isoforms were allowed to hit a single *M*. *musculus* reference [62]. All of the high-scoring segment pairs (HSPs) from each orthologous pair were then used to generate BED files corresponding to COREs between species and a gene-transcript map for the combined analysis that paired *M*. *musculus* gene names to *A*. *cahirinus* isoforms so that gene-level counts could be obtained for the *A*. *cahirinus* assembly. For the *A*. *cahirinus per se* analysis, this gene-transcript map was further augmented with Trinity genes for which orthologs could not be identified so that all transcripts in the *A*. *cahirinus* assembly were quantified.

### Alignment and transcript abundance estimation

Two separate approaches were used to assess transcript abundance in skin, which we will refer to as *per se* and combined. In the *per se* approach, transcript abundance and differential expression analyses were performed within each species independently, i.e. *M*. *musculus* wound versus *M*. *musculus* normal skin. For the *Mus per se* analysis, paired-end and merged reads were mapped to a *M*. *musculus* RefSeq [60] *in silico* transcriptome and transcript abundance estimates were obtained using RSEM [rsem/1.2.28,—paired-end—bowtie2—estimate-rspd—append-names] [63]. For the *Acomys per se* analysis, paired-end and merged reads were mapped to our Trinity transcriptome using the Trinity transcript quantification pipeline [14]. Alignments were performed using Bowtie 2 [bowtie2/2.2.6, -q—phred33—sensitive—dpad 0—gbar 99999999—mp 1,1—np 1—score-min L,0,-0.1 -I 1 -X 1000—no-mixed—no-discordant -p 6 -k 200] [64] before using RSEM [63] to calculate abundance estimates. In the combined approach, merged reads were aligned to the corresponding transcriptome references and then filtered using a custom BED file containing COREs between orthologs. From the resulting count matrices, gene-level abundances were used to evaluate differential expression across samples.

### Differential expression analysis

Analysis of differential expression was performed using the R [65] package voom [66]. All genes were required to contain ≥10 counts-per-million (CPM), based upon the average library size, in three samples (four samples for the *Acomys per se* analysis). The resulting CPM matrices were TMM normalized [67], log transformed and linear model weights were estimated [66]. The resulting log CPM and associated precision weights were then processed using empirical Bayesian moderated t-statistics to determine differentially expressed genes [66]. The previously described transcript quantification approaches, *per se* and combined, resulted in three DE experimental designs–*Mus per se*, *Acomys per se* and *Mus*-*Acomys* combined analyses. Samples were compared in the *per se* models, which analyze differential expression within a single species across the full length of its gene, using pairwise contrasts. The combined model is limited to COREs but allows for a direct comparison between species across the time series.

### BUSCO

Transcriptome quality was assessed using BUSCO [busco/2.0b, -m tran -l vertebrata] [68]. We further assessed the quality of recent transcriptome assemblies in *A*. *cahirinus* [35, 36] and the *M*. *musculus* RefSeq build GRCm38.p3 [60] reference for all transcripts and only those *M*. *musculus* transcripts expressed in our study to make comparisons among assembly qualities.

### Co-expression network analysis

A co-expression network was built using the TMM [67] normalized reads after filtering out low-read-count genes. The resulting matrix contained 14,548 genes and 23 sample columns, where samples included both *M*. *musculus* and *A*. *cahirinus* samples. Gene pairwise correlation was calculated using Spearman correlation in petal [20]. No explicit threshold was designated for correlation cutoff. Instead, petal dynamically selects a threshold that produces a scale-free and small-world network. The scale-free property is mathematically determined such that the node degree distribution follows a power-law distribution, *p_k_* = *Ck*^−*a*^.

While power-law distributions are ubiquitous in nature, problematically, many perceived power-law distributions are fit using linear regression or visually inferred after logarithmically transforming the degree distribution [21], *log*(*p_k_*) = −*a log*(*k*) + *c*. When validating a power-law distribution, petal log transforms the degree distribution so linear regression may be applied. In petal, the slope of the linear regression, which corresponds to the exponent in the power-law function, is expected to lie within the interval (1,3) if the data were derived from a power-law distribution. Also, the R^2^ value is used to determine if the linear fit was good. However, a good R^2^ does not necessarily indicate a good fit, and petal does not perform any explicit hypothesis testing for a power-law distribution. After visualization of the linear fit and residuals, it is clear that there is heteroscedasticity, non-random residuals, heavy tails in the q-q plot of the residuals, and numerous outliers and high leverage points. In linear regression, it is typically assumed that there is homogeneity of variance (homoscedasticity) and residuals are approximately normally distributed. Any null hypothesis significance testing performed using data violating the assumption of homoscedasticity would result in a biased estimate of standard error, which would subsequently affect the p-value and inference. To perform a robust test for a power-law distribution, we tested goodness of fit using bootstrapped maximum likelihood estimation with poweRlaw [0.70.1] [21]) in R [65].

### Pathway analysis

Transcriptional regulators and targets that explain observed gene expression changes in the dataset were identified using IPA where transcriptional regulators may be any molecule capable of affecting the expression of other molecules (e.g. transcription factors, miRNA, kinases or other compounds) [19]. IPA uses manually curated content to identify biologically relevant pathways from a table containing differentially expressed genes, fold changes, FDR, and mean expression. IPA determines if observed changes in expression across genes is generally consistent with changes in any transcriptional regulator expression from the set of all known regulators. Significant regulators are identified using both Fisher’s Exact Test and the manually curated content to determine enrichment for targets of transcriptional regulators. The IPA parameters selected limited inference to experimentally validated, direct and indirect interactions within mammalian species and a maximum of 25 networks per analysis (S6 Table).

Using custom CPython scripts, we subsequently extracted these transcriptional regulators and targets identified by IPA from the co-expression network generated by petal to isolate subnetworks of interest. Each regulator or target was individually isolated from the co-expression network along with neighboring, correlated nodes if those nodes were also transcriptional regulators or targets identified by IPA. This resulted in subnetworks where the primary regulator or target selected was the center of the subnetwork and was connected by an edge (spearman correlation ≥ 0.914 as determined by petal) to every other node in the subnetwork. Subnetworks of all sizes were generated with those including 10 or more nodes identified as being of particular interest. As such, all genes in each subnetwork are differentially expressed, co-expressed and represent either transcriptional regulators or targets. The subnetworks were visualized using Cytoscape [cytoscape/3] [69].

To visually compare expression patterns over time between the two species for subnetworks of particular interest, we used regulators and targets identified within each subnetworks to extract gene-specific expression from our combined *Acomys*-*Mus* expression matrix. We plotted the mean log_2_ expression in line graphs with 95% confidence intervals using custom CPython scripts. As such, genes within line graphs are differentially expressed, co-expressed, and represent transcriptional regulators or targets identified by IPA.

### RT-qPCR analysis

cDNA was generated from 1.5 μg of RNA using SuperScript IV VILO Reverse Transcriptase (Invitrogen 11756050) following the manufacturers protocol. Real-Time PCR was performed using Sso-Fast EvaGreen Supermix (Bio-Rad 172–5200) on a Bio-Rad C1000 Touch Thermal Cycler. Fold change in expression was calculated using the ΔΔCt relative expression method [70]. Sequence of PCR primers can be found in S7 Table.

### Histology

For general histological and immunological studies group sizes were n = 4. For general histology, both wounds and normal skin were fixed in 4% paraformaldehyde overnight, embedded in paraffin wax and sectioned at 10 μm. Masson Trichrome staining was performed following manufacturer’s recommended protocol (Fisher Scientific 22-110-648). For immunocytochemistry on paraffin wax sections the dehydrated sections were microwaved for 4 minutes in citrate buffer pH 6 for antigen retrieval and the Vectastain Eite ABC kit (Vector Labs) was used with diaminobenzidine as the chromogen and the stained sections dehydrated and mounted in Permount. For immunocytochemistry on frozen sections, fixed sections were placed overnight in 30% sucrose at 4°C, embedded in OCT and cryosectioned at 15 μm. Fresh, unfixed tissue was embedded directly in OCT and cryosectioned for *Myh3* and BA-F8 immunostaining. Cryosections were blocked for 1 hr in 0.5% Triton X-100, 0.1% fish skin gelatin (Sigma), 0.1% Tween, 10% donkey serum in phosphate buffered saline (PBS), then incubated with primary antibodies at a 1 in 100 dilution in blocking solution overnight at 4°C. The following day sections were washed in PBS and incubated for 1 hr in the dark with secondary antibodies at a dilution of 1 in 200 and mounted in 80% glycerol containing 1 in 10,000 Hoeschst. The primary antibodies used in this study were *Myh3* (DSHB F1.652), cd206 (Abcam ab64693), Iba-1 (Wako NCNP24), laminin a2 (Abcam ab11576) at 1:100 and myosin heavy chain (DSHB BA-F8) at 1:10. The secondary antibodies used for fluorescence were Alexa Fluor 488 and 647 donkey anti-mouse IgG and Alexa Fluor 488 donkey anti-rabbit IgG (Abcam) at 1:200.

## Supporting information

S1 Fig Transcriptome metric plots. a) BUSCO metrics for individual transcriptome assemblies. *In silico* transcriptome presents all *M*. *musculus* RefSeq transcripts, and *In silico* transcriptome expressed are only the *M*. *musculus* RefSeq sequences expressed in this study. Current study is the *A*. *cahirinus* transcriptome from this study. Mamrot et al. (2017) a multi-tissue, single-replicate *A*. *cahirinus* assembly. Gawriluk et al. (2016) represents an *A*. *cahirinus* ear punch transcriptome generated from time course data b) Top BLAST hits for taxa across Ensembl and SwissProt databases. Taxa had a minimum of 500 hits using *Acomys cahirinus* transcripts as query sequences. c) Multi-dimensional scaling plot of sample gene expression. Abbreviations are formatted as species (aco, mus), time point (0, 7, 14), sample number. Axes represent the leading log fold change across the top two principal axes. d) Ex-N50 plotted against Ex. ExN50 represents the N50 for the highest expressed genes accounting for x% of the total normalized expression. The first point therefore represents a N50 of about 500 for the highest expressed genes representing about 18% of the total normalized expression.
(PDF)

S2 Fig Scale-free and small-world characteristics. A scale-free network follows a power-law distribution with parameters α and x_min_ a) Histogram of alpha values from bootstrapped maximum likelihood estimates b) Histogram of x_min_ values from bootstrapped maximum likelihood estimates. A network with small-world characteristics has c) large average clustering coefficient and d) short average path length.
(PDF)

S3 Fig *A*. *cahirinus* early wound dermis shows limited *Iba-1* +ve cells by immunofluorescence. Immunofluorescence was performed on fixed cryosectioned tissues for *Iba-1* on (**a**) day 7, (**b**) day 10, (**c**) day 14 and (**d**) day 18 wound sections. Green–*Iba-1*; Blue–Hoechst. Scale bars 100 μm.
(PDF)

S4 Fig Linear fit assessment for power-law distribution. a) Linear fit of log frequency-log degree plot. b) Residual plot from the log frequency-log degree fit. c) Q-Q plot demonstrating deviations of residual plot from a normal distribution. d) Studentized residuals beyond an absolute value of 2 are labeled as outliers (red plus). High leverage points are indicated by green xs.
(PDF)

S5 Fig Maximum likelihood estimates across bootstrapped datasets. Estimated parameters include x_min_ and α (Par 1). Corresponding p-values are also generated for bootstrapped estimates where the null is a power-law distribution. Red lines indicate 95% confidence intervals.
(PDF)

S6 Fig Factor plots of log2(RNA-Seq expression + 1) across samples. Plot of *Il10* expression where day 0 *A*. *cahirinus* expression is low/zero. Bars represent 95% confidence intervals.
(PDF)

S1 Table Raw read counts. (PDF)

S2 Table Trimmed read counts. (PDF)

S3 Table Trinity statistics. (PDF)

S4 Table Counts for differentially expressed genes across contrasts. FDR represents the false discovery rate, and FC represents the fold change. Known represents *A*. *cahirinus* genes with orthologs identified in *M*. *musculus*. Unknown represents genes without an identifiable annotation.
(PDF)

S5 Table Threshold statistics from petal. Correlation thresholds were determined using scale-free parameters R^2^ and the slope of the log-log plot as well as small-world parameters mean clustering coefficient (MeanCC) and mean path length (MeanPath). The %Used represents the percent of the original dataset in the network and %BigComp provides the percent of network vertices in the largest component.
(PDF)

S6 Table IPA parameters. (PDF)

S7 Table RT-qPCR oligo sequences. List of RT-qPCR oligos used for both *Mus* and *Acomys*.
(PDF)

S8 Table Petal R session information. R environment session information for petal analysis.
(PDF)

S1 Data These data represent the output from IPA’s Bio Functions within the CORE Analysis which provides detailed examination of downstream gene effects and their significance. Evidence is derived from published literature and significance is determined from a provided time-course differential expression days 7–0 contrast results. These data were specifically derived from regeneration-associated genes as identified by IPA.
(TXT)

S2 Data These data represent the output from IPA’s Bio Functions within the CORE Analysis which provides detailed examination of downstream gene effects and their significance. Evidence is derived from published literature and significance is determined from a provided time-course differential expression days 14–0 contrast results. These data were specifically derived from regeneration-associated genes as identified by IPA.
(TXT)

S3 Data These data represent the output from IPA’s regulatory effects analysis for the time-course 7–0 contrast where IPA attempts to predict activated or inhibited upstream regulators that may increase or decrease phenotypic or functional outcomes downstream. The consistency score is calculated by IPA for each regulator effect network. The consistency score is higher for networks that are directionally consistent, that is where most of the expression states for regulators and targets match expected states for given diseases or functions based upon existing literature. Identified regulators and targets are provided along with their predicted function.
(TXT)

S4 Data These data represent the output from IPA’s regulatory effects analysis for the time-course 14–0 contrast where IPA attempts to predict activated or inhibited upstream regulators that may increase or decrease phenotypic or functional outcomes downstream. The consistency score is calculated by IPA for each regulator effect network. The consistency score is higher for networks that are directionally consistent, that is where most of the expression states for regulators and targets match expected states for given diseases or functions based upon existing literature. Identified regulators and targets are provided along with their predicted function.
(TXT)

S5 Data *Mus per se–*Day 7 vs Day 0 differential expression results table. Column A–fold change in expression. Column B–official gene symbol. Column C–official gene name. Column D—Benjamini and Hochberg false discover rate adjustment. Column E–log fold change in expression. Column F–average expression of gene across samples. Column G–t statistic. Column H–p-value. Column I–B statistic (log odds). Column J–log counts per million of expression across samples.
(XLSX)

S6 Data *Mus per se–*Day 14 vs Day 0 differential expression results table. Column headers as described in S5 Data.
(XLSX)

S7 Data *Acomys per se–*Day 7 vs Day 0 differential expression results table. Column headers as described in S5 Data.
(XLSX)

S8 Data *Acomys per se–*Day 14 vs Day 0 differential expression results table. Column headers as described in S5 Data.
(XLSX)

S9 Data Combined Analyses–*Acomys* vs *Mus*–Day 7 vs Day 0 differential expression results table. Column headers as described in S5 Data.
(XLSX)

S10 Data Combined Analyses–*Acomys* vs *Mus*–Day 14 vs Day 0 differential expression results table. Column headers as described in S5 Data.
(XLSX)

S11 Data IPA results. Results from IPA core analyses. Each sheet represents the contrasts for S1–S4 Data.
(XLSX)
