# Supplementary material for: Comparative transcriptomic analysis of dermal wound healing reveals de novo skeletal muscle regeneration in Acomys cahirinus
Source: PLoS One. 2019 May 29;14(5):e0216228. doi: 10.1371/journal.pone.0216228 (PMC6541261; doi:10.1371/journal.pone.0216228)
Supplement: S1 Fig — a) BUSCO metrics for individual transcriptome assemblies. In silico transcriptome presents all M. musculus RefSeq transcripts, and In silico transcriptome expressed are only the M. musculus RefSeq sequences expressed in this study. Current study is the A. cahirinus transcriptome from this study. Mamrot et al. (2017) a multi-tissue, single-replicate A. cahirinus assembly. Gawriluk et al. (2016) represents an A. cahirinus ear punch transcriptome generated from time course data b) Top BLAST hits for taxa across Ensembl and SwissProt databases. Taxa had a minimum of 500 hits using Acomys cahirinus transcripts as query sequences. c) Multi-dimensional scaling plot of sample gene expression. Abbreviations are formatted as species (aco, mus), time point (0, 7, 14), sample number. Axes represent the leading log fold change across the top two principal axes. d) Ex-N50 plotted against Ex. ExN50 represents the N50 for the highest expressed genes accounting for x% of the total normalized expression. The first point therefore represents a N50 of about 500 for the highest expressed genes representing about 18% of the total normalized expression. (PDF) [file pone.0216228.s001.pdf]

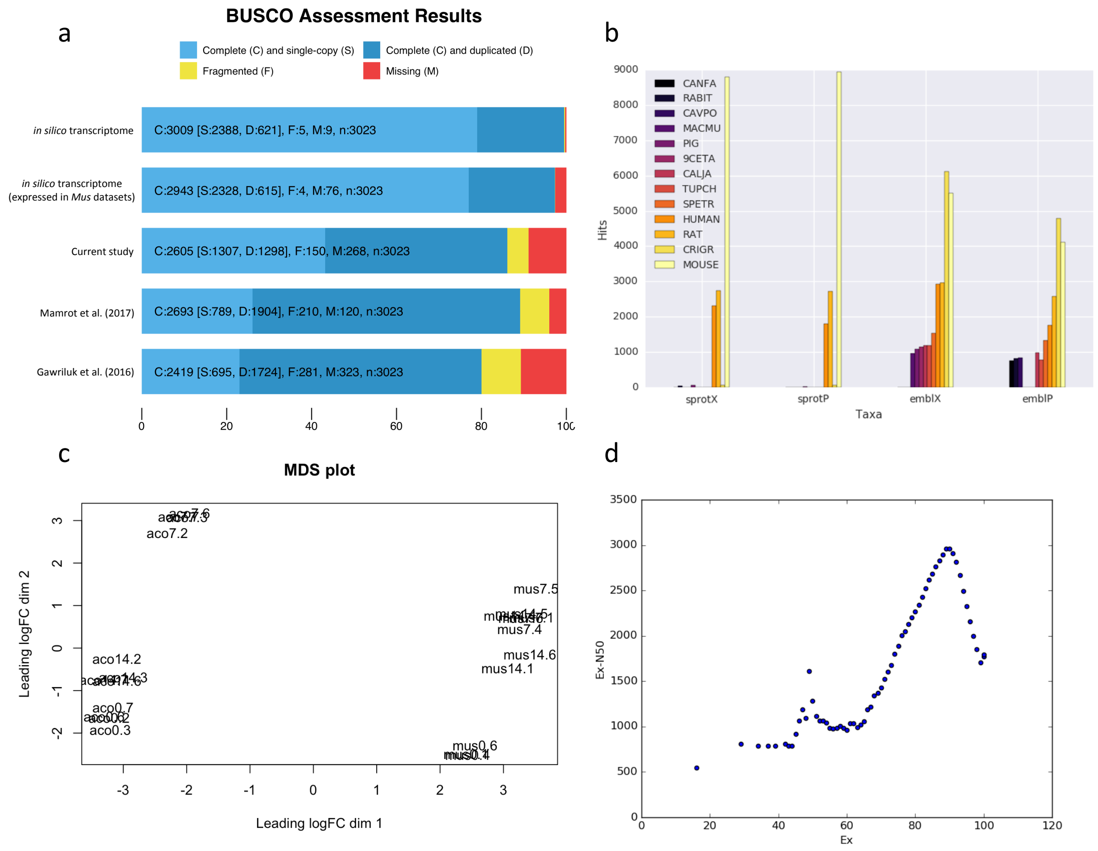


**Supplementary Figure 1. Transcriptome metric plots.** a) BUSCO metrics for individual transcriptome assemblies. *In silico* transcriptome presents all *Mus* RefSeq transcripts, and *In silico* transcriptome expressed are only the *Mus* RefSeq sequences expressed in this study. Current study is the *Acomys* transcriptome from this study. Mamrot et al. (2017) a multi-tissue, single-replicate *Acomys* assembly. Gawriluk et al. (2016) represents an *Acomys* ear punch transcriptome generated from time course data b) Top BLAST hits for taxa across Ensembl and SwissProt databases. Taxa had a minimum of 500 hits using *Acomys cahirinus* transcripts as query sequences. c) Multi-dimensional scaling plot of sample gene expression. Abbreviations are formatted as species (aco, mus), time point (0, 7, 14), sample number. Axes represent the leading log fold change across the top two principal axes. d) Ex-N50 plotted against Ex. ExN50 represents the N50 for the highest expressed genes accounting for x% of the total normalized expression. The first point therefore represents a N50 of about 500 for the highest expressed genes representing about 18% of the total normalized expression.
