# Supplementary material for: Comparative transcriptomic analysis of dermal wound healing reveals de novo skeletal muscle regeneration in Acomys cahirinus
Source: PLoS One. 2019 May 29;14(5):e0216228. doi: 10.1371/journal.pone.0216228 (PMC6541261; doi:10.1371/journal.pone.0216228)
Supplement: S2 Fig — A scale-free network follows a power-law distribution with parameters α and xmin a) Histogram of alpha values from bootstrapped maximum likelihood estimates b) Histogram of xmin values from bootstrapped maximum likelihood estimates. A network with small-world characteristics has c) large average clustering coefficient and d) short average path length. (PDF) [file pone.0216228.s002.pdf]

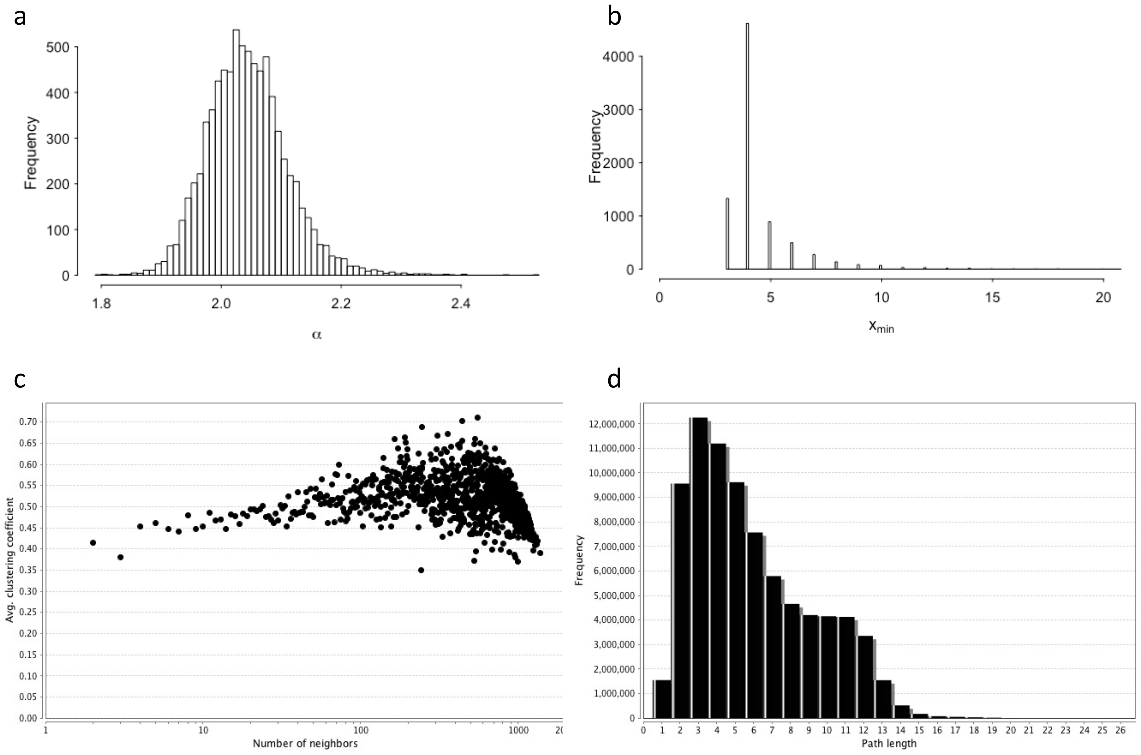


**S2 Fig. Scale-free and small-world characteristics.** A scale-free network follows a power-law distribution with parameters α and $x_{min}$ a) Histogram of alpha values from bootstrapped maximum likelihood estimates b) Histogram of $x_{min}$ values from bootstrapped maximum likelihood estimates. A network with small-world characteristics has c) large average clustering coefficient and d) short average path length
