# Supplementary material for: Comparative transcriptomic analysis of dermal wound healing reveals de novo skeletal muscle regeneration in Acomys cahirinus
Source: PLoS One. 2019 May 29;14(5):e0216228. doi: 10.1371/journal.pone.0216228 (PMC6541261; doi:10.1371/journal.pone.0216228)
Supplement: S3 Fig — Immunofluorescence was performed on fixed cryosectioned tissues for Iba-1 on (a) day 7, (b) day 10, (c) day 14 and (d) day 18 wound sections. Green–Iba-1; Blue–Hoechst. Scale bars 100 μm. (PDF) [file pone.0216228.s003.pdf]

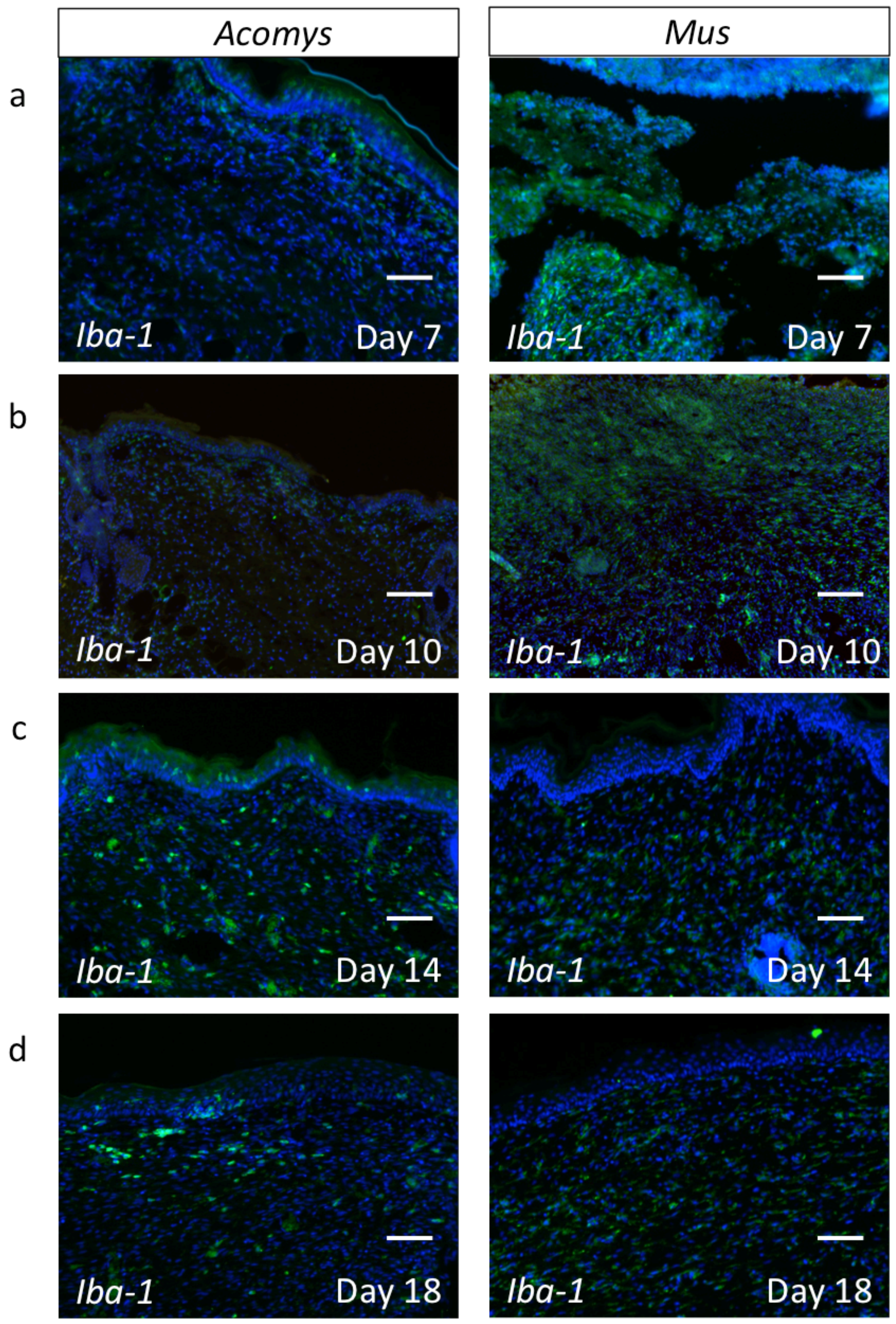

S4 Fig. *Acomys* early wound dermis shows limited *Iba-1* +ve cells by immunofluorescence. Immunofluorescence was performed on fixed cryosectioned tissues for *Iba-1* on (**a**) day 7, (**b**) day 10, (**c**) day 14 and (**d**) day 18 wound sections. Green – *Iba-1*; Blue – DAPI. Scale bars 100 mm
