# Supplementary material for: Comparative transcriptomic analysis of dermal wound healing reveals de novo skeletal muscle regeneration in Acomys cahirinus
Source: PLoS One. 2019 May 29;14(5):e0216228. doi: 10.1371/journal.pone.0216228 (PMC6541261; doi:10.1371/journal.pone.0216228)
Supplement: S4 Fig — a) Linear fit of log frequency-log degree plot. b) Residual plot from the log frequency-log degree fit. c) Q-Q plot demonstrating deviations of residual plot from a normal distribution. d) Studentized residuals beyond an absolute value of 2 are labeled as outliers (red plus). High leverage points are indicated by green xs. (PDF) [file pone.0216228.s004.pdf]

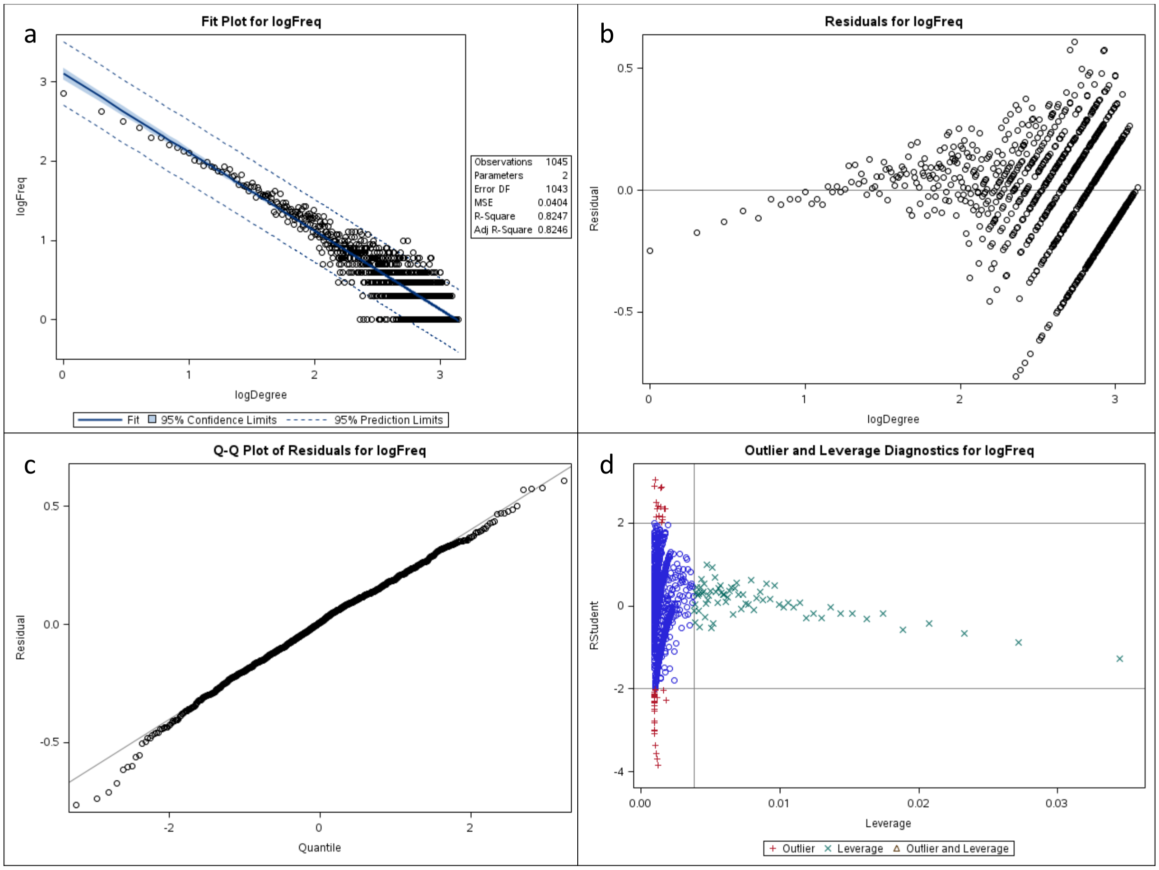

S5 Fig. Linear fit assessment for power-law distribution. a) Linear fit of log frequency-log degree plot. b) Residual plot from the log frequency-log degree fit. c) Q-Q plot demonstrating deviations of residual plot from a normal distribution. d) Studentized residuals beyond an absolute value of 2 are labeled as outliers (red plus). High leverage points are indicated by green xs.
