# Supplementary material for: Comparative transcriptomic analysis of dermal wound healing reveals de novo skeletal muscle regeneration in Acomys cahirinus
Source: PLoS One. 2019 May 29;14(5):e0216228. doi: 10.1371/journal.pone.0216228 (PMC6541261; doi:10.1371/journal.pone.0216228)
Supplement: S5 Fig — Estimated parameters include xmin and α (Par 1). Corresponding p-values are also generated for bootstrapped estimates where the null is a power-law distribution. Red lines indicate 95% confidence intervals. (PDF) [file pone.0216228.s005.pdf]

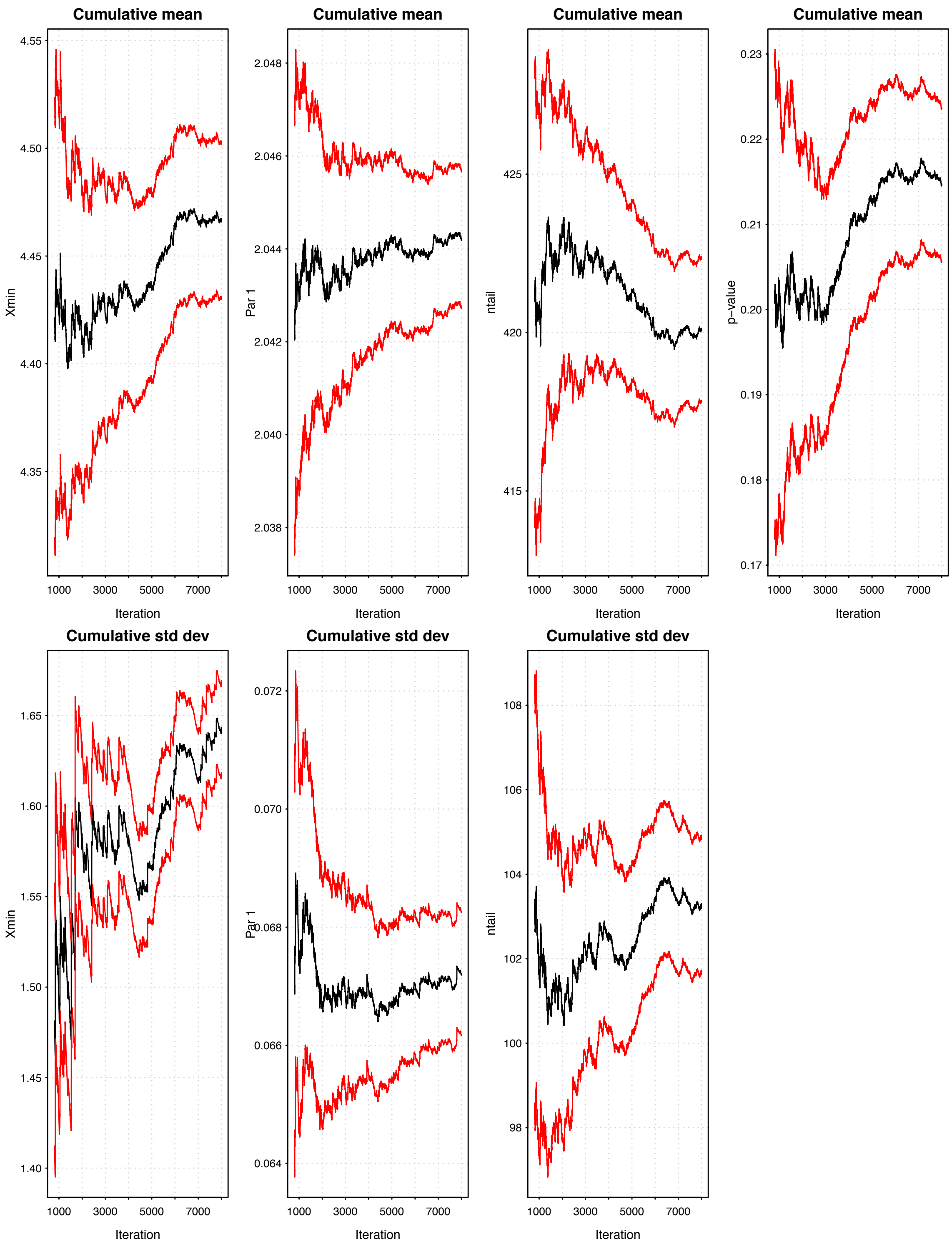

S6 Fig. Maximum likelihood estimates across bootstrapped datasets. Estimated parameters include x_{min} and α (Par 1). Corresponding p-values are also generated for bootstrapped estimates where the null is a power-law distribution. Red lines indicate 95% confidence intervals.
