# Supplementary material for: Comparative transcriptomic analysis of dermal wound healing reveals de novo skeletal muscle regeneration in Acomys cahirinus
Source: PLoS One. 2019 May 29;14(5):e0216228. doi: 10.1371/journal.pone.0216228 (PMC6541261; doi:10.1371/journal.pone.0216228)
Supplement: S6 Fig — Plot of Il10 expression where day 0 A. cahirinus expression is low/zero. Bars represent 95% confidence intervals. (PDF) [file pone.0216228.s006.pdf]

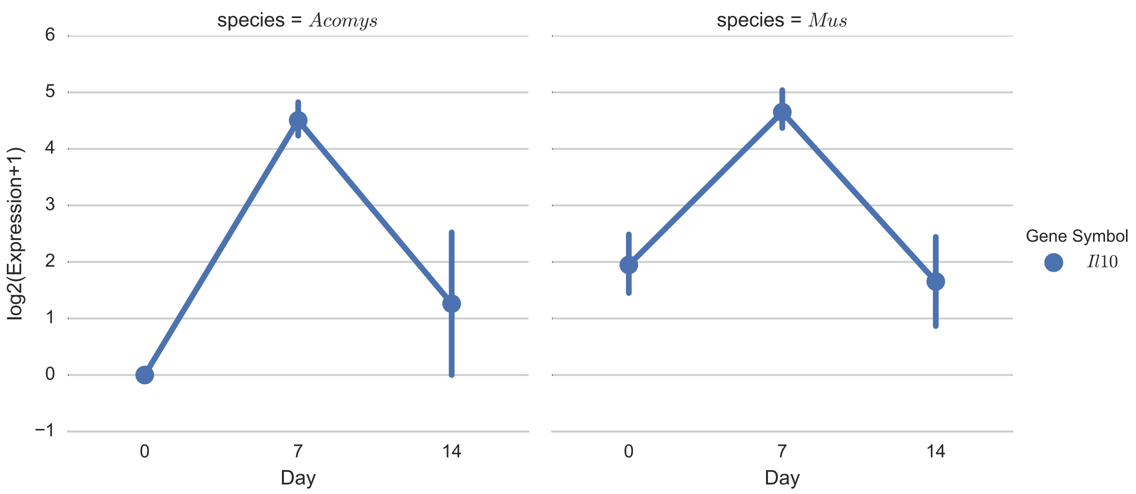

S7 Fig. Factor plots of log2(RNA-Seq expression + 1) across samples. Plot of Il10 expression where day 0 *Acomys* expression is low/zero.
