# Supplementary material for: Comparative transcriptomic analysis of dermal wound healing reveals de novo skeletal muscle regeneration in Acomys cahirinus
Source: PLoS One. 2019 May 29;14(5):e0216228. doi: 10.1371/journal.pone.0216228 (PMC6541261; doi:10.1371/journal.pone.0216228)
Supplement: S1 Table — (PDF) [file pone.0216228.s007.pdf]

Supplementary Table 1. Raw read counts.

Sample	Read pairs
AcomysDay0-2	45,122,164
AcomysDay0-3	39,872,870
AcomysDay0-6	40,147,150
AcomysDay0-7	41,168,457
AcomysDay14-2	44,291,071
AcomysDay14-3	39,048,479
AcomysDay14-6	43,237,450
AcomysDay14-7	44,115,913
AcomysDay7-2	45,592,500
AcomysDay7-3	37,313,402
AcomysDay7-6	43,080,314
AcomysDay7-7	39,985,139
DSN	36,249,069
musDay0-1	41,395,798
musDay0-4	46,868,984
musDay0-5	35,401,757
musDay0-6	47,018,171
musDay14-1	39,399,711
musDay14-4	40,156,144
musDay14-5	39,842,678
musDay14-6	46,674,222
musDay7-1	49,661,108
musDay7-4	42,802,619
musDay7-5	44,198,536
musDay7-6	36,957,099
