# Supplementary material for: Comparative transcriptomic analysis of dermal wound healing reveals de novo skeletal muscle regeneration in Acomys cahirinus
Source: PLoS One. 2019 May 29;14(5):e0216228. doi: 10.1371/journal.pone.0216228 (PMC6541261; doi:10.1371/journal.pone.0216228)
Supplement: S2 Table — (PDF) [file pone.0216228.s008.pdf]

Supplementary Table 2. Trimmed read counts.

Sample	Read Pairs
AcomysDay0-2	30,789,592
AcomysDay0-3	27,277,227
AcomysDay0-6	24,506,722
AcomysDay0-7	30,548,410
AcomysDay14-2	30,432,360
AcomysDay14-3	26,768,258
AcomysDay14-6	29,928,401
AcomysDay14-7	30,723,215
AcomysDay7-2	31,048,454
AcomysDay7-3	24,725,243
AcomysDay7-6	29,425,179
AcomysDay7-7	27,780,754
DSN	24,363,676
musDay0-1	27,749,562
musDay0-4	32,015,683
musDay0-5	21,304,486
musDay0-6	33,160,442
musDay14-1	27,292,598
musDay14-4	27,588,537
musDay14-5	27,516,539
musDay14-6	32,563,402
musDay7-1	33,330,758
musDay7-4	29,131,200
musDay7-5	31,201,338
musDay7-6	25,816,793
