# Supplementary material for: Comparative transcriptomic analysis of dermal wound healing reveals de novo skeletal muscle regeneration in Acomys cahirinus
Source: PLoS One. 2019 May 29;14(5):e0216228. doi: 10.1371/journal.pone.0216228 (PMC6541261; doi:10.1371/journal.pone.0216228)
Supplement: S3 Table — (PDF) [file pone.0216228.s009.pdf]

Supplementary Table 3. Trinity statistics

################################					
##	Counts of transcripts, etc.				
################################					
Total	trinity	'genes':		220101	
Total	trinity	transcripts:		283780	
Percent	GC:		47.12		
##					
##	Stats based on all transcript contigs:				
##					
	Contig	N10:		5762	
	Contig	N20:		4110	
	Contig	N30:		3092	
	Contig	N40:		2360	
	Contig	N50:		1794	
	Median	contig	length:		911
	Average	contig:		1408.38	
	Total	assembled	bases:		399669981
###					
##	Stats based on only longest isoform per Trinity gene:				
###					
	Contig	N10:		4225	
	Contig	N20:		2677	
	Contig	N30:		1900	
	Contig	N40:		1456	
	Contig	N50:		1170	
	Median	contig	length:		804
	Average	contig:		1112.41	
	Total	assembled	bases:		244842727
