# Supplementary material for: Comparative transcriptomic analysis of dermal wound healing reveals de novo skeletal muscle regeneration in Acomys cahirinus
Source: PLoS One. 2019 May 29;14(5):e0216228. doi: 10.1371/journal.pone.0216228 (PMC6541261; doi:10.1371/journal.pone.0216228)
Supplement: S4 Table — FDR represents the false discovery rate, and FC represents the fold change. Known represents A. cahirinus genes with orthologs identified in M. musculus. Unknown represents genes without an identifiable annotation. (PDF) [file pone.0216228.s010.pdf]

Supplementary Table 4. Counts for differentially expressed genes across contrasts.

	Total	FDR	FC	Known	Unknown
Acomys 7-0	21,379	11,279	9,254	6,478	2,776
Acomys 14-0	21,379	6,408	5,050	3,735	1,315
Mus 7-0	14,677	8,849	6,095	-	-
Mus 14-0	14,677	7,146	4,625	-	-
Time series 7-0	14,548	5,074	4,172	-	-
Time series 14-0	14,548	3,556	2,977	-	-

FDR represents the number of loci ≤ 0.05 false discovery rate, and FC represents the number of loci with fold change ≥ ±1.5. Known represents *Acomys* genes with orthologs identified in *Mus*. Unknown represents genes without an identifiable annotation.
