# Supplementary material for: Comparative transcriptomic analysis of dermal wound healing reveals de novo skeletal muscle regeneration in Acomys cahirinus
Source: PLoS One. 2019 May 29;14(5):e0216228. doi: 10.1371/journal.pone.0216228 (PMC6541261; doi:10.1371/journal.pone.0216228)
Supplement: S5 Table — Correlation thresholds were determined using scale-free parameters R2 and the slope of the log-log plot as well as small-world parameters mean clustering coefficient (MeanCC) and mean path length (MeanPath). The %Used represents the percent of the original dataset in the network and %BigComp provides the percent of network vertices in the largest component. (PDF) [file pone.0216228.s011.pdf]

Supplementary Table 5. Threshold statistics from petal.

Threshold	R^2	Slope	MeanCC	MeanPath	%Used	%BigComp
0.974	0.9135	-1.8863	0.3638	7.2287	19.1092	65
0.959	0.9135	-1.4742	0.4065	6.8608	38.9057	81.8198
0.944	0.8997	-1.2678	0.4464	7.644	55.8015	93.9148
0.929	0.8678	-1.1518	0.4604	6.6075	68.2568	95.6495
0.914	0.8352	-1.0458	0.4806	5.8879	76.7391	96.8112
0.899	0.7966	-0.9531	0.5016	5.4539	82.843	98.0252
0.884	0.7471	-0.8754	0.5189	4.9425	87.3178	98.8664

Correlation thresholds were determined using scale-free parameters R^2 and the slope of the log-log plot as well as small-world parameters mean clustering coefficient (MeanCC) and mean path length (MeanPath). The %Used represents the percent of the original dataset in the network and %BigComp provides the percent of network vertices in the largest component.
