# Supplementary material for: Comparative transcriptomic analysis of dermal wound healing reveals de novo skeletal muscle regeneration in Acomys cahirinus
Source: PLoS One. 2019 May 29;14(5):e0216228. doi: 10.1371/journal.pone.0216228 (PMC6541261; doi:10.1371/journal.pone.0216228)
Supplement: S6 Table — (PDF) [file pone.0216228.s012.pdf]

Supplementary Table 6. IPA general settings

Reference set	
	Ingenuity Knowledge Base (Genes Only)
Relationships to consider	
	Direct and Indirect
Networks	
	35 Molecules per network
	25 Networks per analysis
Node Types	
	complex
	cytokine
	enzyme
	G-protein coupled receptor
	group
	growth factor
	ion channel
	kinase
	ligand-dependent nuclear receptor
	mature miRNA
	miRNA
	peptidase
	phosphatase
	transcription regulator
	translation regulator
	transmembrance receptor
	transporter
	other
Data Sources	
	All
Confidence	
	Experimentally observed
Species	
	All
Tissues & Cell Lines	
	None
Mutation	
	All
