# Supplementary material for: Comparative transcriptomic analysis of dermal wound healing reveals de novo skeletal muscle regeneration in Acomys cahirinus
Source: PLoS One. 2019 May 29;14(5):e0216228. doi: 10.1371/journal.pone.0216228 (PMC6541261; doi:10.1371/journal.pone.0216228)
Supplement: S7 Table — List of RT-qPCR oligos used for both Mus and Acomys. (PDF) [file pone.0216228.s013.pdf]

S7 Table. RT-qPCR oligo sequences.

Acomys RT Primers

Gene	Forward Primer Sequence	Reverse Primer Sequence
Myog	CTTTTCTTCCAGGGGACCTC	ACCAGGAGCCCCACTTCTAT
Myf5	CAGCTCAGCTTTGTGTGCTC	GCTGCCAGTTCTCACCTTCT
MyoD1	TCTGGCCAAGCGACTCTTAT	TCCAGTCCTGCGTGTAACAG
Myh3	ATGTCGATGGAAGCCAAGAG	GGTTCCCCAACAGGATTCTC
Col6a1	CGTTGATGAACTCCATGCTG	AGGATGTGCGTGTAGCTGTG
Col12a1	GTGCGCAAAACATCTCAGAA	CGTACAATGGGCAAGGCTAT
Wnt6	ATGGAACAGGCCTGAGTGAC	TGGAACTGCTCCAGTCACAG
Wnt7a	CACACCCACCACTTTCACTG	AGCAGAACAGACCAGCCACT
Wnt2	CATGTAGGGGTCGTCCAGTC	CGGATGACCAAGTGTGAGTG
Il-10	TCCTGAGGGTCTTCAGCTTC	GGTTGCCAAGCCTTATCAGA
Gapdh	CGACCTTCACCATCTTGTCA	CCCACCAACCTGGTTCCTAT

Mus RT Primers

Gene	Forward Primer Sequence	Reverse Primer Sequence
Myog	ACCAGGAGCCCCACTTCTAT	GTCCCCAGTCCCTTTTCTTC
Myf5	CAGCTCAGCTTTGTGTGCTC	GCCAGTTCTCCCCTTCTGAG
MyoD1	GCCACTCAGGTCTCAGGTGT	GCTCTGGCCAAGCAACTCT
Myh3	ATGAGTAGCGACACCGAGATG	ACAAAGCAGTAGGTTTTGGCAT
Col6a1	TTGCCGTCTGAAAACAACAG	CAGCATGGATTTCATCAACG
Col12a1	TGCACCTGAGCCATAGAGTG	ATTCAAGGCCTTCCGAATTT
Wnt6	CTCCTACAGTGTGGTTGTCAGG	GCGCATCCATAAAGAGTCTTGA
Wnt7a	TGAACTTACACAATAACGAGGCG	GTGGTCCAGCACGTCTTAGT
Wnt2	CTCGGTGGAATCTGGCTCTG	CACATTGTCACACATCACCCT
Il-10	GCTCTTACTGACTGGCATGAG	CGCAGCTCTAGGAGCATGTG
Gapdh	TGACCTCAACTACATGGTCTACA	CTTCCCATTCTCGGCCTTG
