# Supplementary material for: Comparative transcriptomic analysis of dermal wound healing reveals de novo skeletal muscle regeneration in Acomys cahirinus
Source: PLoS One. 2019 May 29;14(5):e0216228. doi: 10.1371/journal.pone.0216228 (PMC6541261; doi:10.1371/journal.pone.0216228)
Supplement: S8 Table — R environment session information for petal analysis. (PDF) [file pone.0216228.s014.pdf]

S8 Table. Petal R Session Information

```
R version 3.3.1 (2016-06-21)
Platform: x86_64-apple-darwin13.4.0 (64-bit)
Running under: OS X 10.10.5 (Yosemite)

locale:
[1] en_US.UTF-8/en_US.UTF-8/en_US.UTF-8/C/en_US.UTF-8/en_US.UTF-8

attached base packages:
[1] stats     graphics  grDevices utils     datasets  methods   base

other attached packages:
[1] petal_0.1.5

loaded via a namespace (and not attached):
[1] tools_3.3.1
```
